# MEK2 Is Sufficient but Not Necessary for Proliferation and Anchorage-Independent Growth of SK-MEL-28 Melanoma Cells

**DOI:** 10.1371/journal.pone.0017165

**Published:** 2011-02-18

**Authors:** Chih-Shia Lee, Karl J. Dykema, Danielle M. Hawkins, David M. Cherba, Craig P. Webb, Kyle A. Furge, Nicholas S. Duesbery

**Affiliations:** 1 Laboratory of Cancer and Developmental Cell Biology, Van Andel Research Institute, Grand Rapids, Michigan, United States of America; 2 Graduate Program in Biochemistry and Molecular Biology, Michigan State University, East Lansing, Michigan, United States of America; 3 Laboratory of Computational Biology, Van Andel Research Institute, Grand Rapids, Michigan, United States of America; 4 Program of Translational Medicine, Van Andel Research Institute, Grand Rapids, Michigan, United States of America; Dana-Farber Cancer Institute, United States of America

## Abstract

Mitogen-activated protein kinase kinases (MKK or MEK) 1 and 2 are usually treated as redundant kinases. However, in assessing their relative contribution towards ERK-mediated biologic response investigators have relied on tests of necessity, not sufficiency. In response we developed a novel experimental model using lethal toxin (LeTx), an anthrax toxin-derived pan-MKK protease, and genetically engineered protease resistant MKK mutants (MKKcr) to test the sufficiency of MEK signaling in melanoma SK-MEL-28 cells. Surprisingly, ERK activity persisted in LeTx-treated cells expressing MEK2cr but not MEK1cr. Microarray analysis revealed non-overlapping downstream transcriptional targets of MEK1 and MEK2, and indicated a substantial rescue effect of MEK2cr on proliferation pathways. Furthermore, LeTx efficiently inhibited the cell proliferation and anchorage-independent growth of SK-MEL-28 cells expressing MKK1cr but not MEK2cr. These results indicate in SK-MEL-28 cells MEK1 and MEK2 signaling pathways are not redundant and interchangeable for cell proliferation. We conclude that in the absence of other MKK, MEK2 is sufficient for SK-MEL-28 cell proliferation. MEK1 conditionally compensates for loss of MEK2 only in the presence of other MKK.

## Introduction

The mitogen-activated protein kinase (MAPK) kinase (MEK) pathway is highly activated in many cancers including melanoma. Indeed, more than eighty percent of human melanomas harbor somatic B-Raf or N-Ras mutations causing constitutive activation of MEK1 and 2 [1], [2]. Elevated MEK1 and 2 activities promote melanoma tumorigenesis, angiogenesis, and progression [3], [4], [5], [6], [7]. Conversely, biological and small molecule inhibitors targeting MEK 1 and 2 inhibit melanoma cell proliferation and xenograft tumor growth as well as metastasis [8], [9], [10], [11]. Consequently, a great deal of effort has been placed on developing inhibitors of MEK1 and 2 for therapeutic purposes [12]. Despite this, MEK1/2 inhibitors have failed to meet expectations in clinical trials owing to poor efficacy and unexpected toxicities [13], [14], [15]. A better understanding of the relative contributions of MEK1 and 2 may aid in the design of more effective therapies for treating melanoma and other MEK-dependent cancers.

MEK1 and 2 share greater than 85% amino acid homology in their kinase domains. They are considered to have overlapping and redundant functions since their only known substrates are the mitogen-activated/extracellular signal-regulated protein kinase (MAPK or ERK) 1 and 2. This conclusion is borne out by studies showing inhibition of either MEK1 or MEK2 has no effect on cell proliferation and combined inhibition of these kinases is required before an effect is observed [16]. However, interpretation of these results is confounded by evidence suggesting MEK1 and MEK2 are not redundant. For instance, MEK1 knockout mice are embryonic lethal [17]. However, knockout of MEK2 has no effect on mouse viability [18], suggesting that MEK1 and MEK2 are differentially expressed during mouse embryogenesis. Still, others have shown that shRNA-mediated MEK2 knockdown has much stronger inhibitory effect on cell proliferation than MEK1 [19], and in another context that MEK1 but not MEK2 is required for experimentally-induced benign epidermal papilloma formation [20]. However, in assessing the relative contribution of MEK1 and MEK2 towards ERK-mediated biologic response each of these studies has relied on tests of necessity, but not sufficiency. Since independent observations have suggested cross talk between MKK signaling pathways [21], [22], [23], redundancy of MEK1 and MEK2 may be dependent on co-operations of other MKK members, and the difference between MEK1 and MEK2 may not be revealed until the co-operations from other MKK members are blocked.

We designed two series of experiments to test the hypothesis that the functions of MEK1 and MEK2 in SK-MEL-28 human melanoma cells are critical and interchangeable for melanoma cell proliferation. In the first experiment we used a traditional siRNA-based approach to determine the necessity of MEK1 and MEK2 signaling pathway for SK-MEL-28 cell proliferation. In this experiment either MEK1 or MEK2 was knocked-down by the specific siRNA while the other MEK isoform and other MKK family proteins were expressed. In the second experiment we developed a novel experimental system that allowed us to evaluate the sufficiency of individual MEK signaling pathways for cell proliferation. To do this, we took advantage of the MEK/MKK-specific proteolytic activity of anthrax lethal toxin (LeTx) [24]. LeTx is a binary toxin secreted by the bacterium *Bacillus anthracis*. It is composed of a binding moiety called protective antigen (PA) and an enzymatic moiety, lethal factor (LF). During cellular intoxication, PA binds to the anthrax receptors expressed on the host cell surface and then is cleaved by a furin-like protease. This cleavage removes a 20-kDa N-terminal fragment and leaves a 63-kDa C-terminal truncation, PA_63_, on the cell surface. PA_63_ then forms an oligomerized channel to which the LF binds. After the binding of LF, the complex is internalized into cells through the endosomal internalization pathway. The acidic environment of the endosome causes a conformational change in PA_63_ that results in the formation of pores through which LF is released into cytosol (reviewed in [25]).

Since its initial identification as an MEK1/2-specific protease [24], additional members of the MAP kinase kinase family, including MKK3, 4, 6 and 7 but not MEK5, have also been found to be substrates of LF ([26], [27], [28] and discussed in [29]). LF specifically cleaves the N-terminus of MEK and other MKK proteins at the consensus cleavage site: three to four basic or proline residues followed by three variable residues followed by an aliphatic residue: (B/P)_3–4_-(X)_3_-Al. The N-termini of this family of proteins harbor MAP kinase docking sites that are required for the interaction between MEK/MKK and MAPK [30]. Not surprisingly, after LF cleavage, the C-terminal part of MEK/MKK, which contains the kinase domain, loses its affinity for the downstream MAP kinases [31], [32]. In addition, biochemical evidence has shown that loss of the amino terminus may destabilize MEK, leading to the decreased intrinsic kinase activity that is observed following LF-mediated proteolysis [31]. Thus, cleavage of MEK/MKK by LF results in a blockade of not only the ERK pathway but also of the p38 MAPK and JNK pathways.

We used LeTx to inhibit both MEK1 and MEK2 as well as other MKK signaling pathways in SK-MEL-28 cells, and then selectively rescued either MEK1 or MEK2 signaling by expressing a cleavage-resistant form of MEK (MEKcr).

The results of these experiments lead us to conclude that in the absence of other MKK, MEK2 is sufficient to drive SK-MEL-28 cell proliferation. MEK1 can conditionally compensate for loss of MEK2 only in the presence of other MKK. Thus, our findings indicate MEK1 and MEK2 signaling pathways possess non-redundant biologic activities.

## Results

### Necessity of Individual MEK Signaling for Melanoma Cell Proliferation

**Figure 1:**
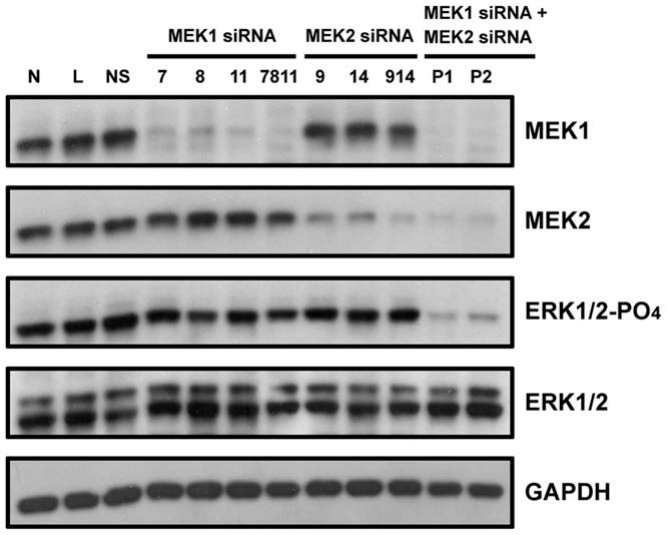
Necessity of MEK1 and MEK2 signaling pathways for ERK activation in SK-MEL-28 cells. SK-MEL-28 cells were transfected with nothing (N), lipid only (L), non-silencing control siRNA (NS), MEK-specific siRNA (MEK1 siRNA 7, 8, 11, and MEK2 siRNA 9, and 14), pools of MEK-specific siRNA (MEK1 siRNA 7+8+11 and MEK2 siRNA 9+14) or combinations of pools of MEK1- and MEK2-specific siRNA (P1, MEK1 siRNA 8+11 plus MEK2 siRNA 9+14; P2, MEK1 siRNA 7+8+11 plus MEK2 siRNA 9+14) as described in Material and Methods. After transfection, cells were trypsinized and split into separate dishes for cell lysate collection and for cell cycle analysis (results shown in Table 1). Seventy-two hours later, whole cell lysates were harvested and immunoblotted. The efficiency of siRNA-mediated MEK knock down was examined by immunoblotting with antibodies against MEK1 (top panel) or MEK2 (the second panel). ERK activation was detected by antibodies against phosphorylated ERK (the third panel). Total ERK expression was detected by ERK antibody as a control (the fourth panel). Antibody against GAPDH was used as a loading control (bottom panel).

To test the hypothesis that MEK1 and MEK2 are redundant and interchangeable for melanoma cell proliferation, we first evaluated the necessity of MEK1 or MEK2 signaling for melanoma cell proliferation by siRNA-mediated knockdown. To do this, we transfected human melanoma SK-MEL-28 cells with individual siRNAs or pools of siRNAs specifically targeting either MEK1 or MEK2. SK-MEL-28 cells harbor the B-Raf_V600E mutation [1], which promotes constitutive activation of MEK1 and MEK2, and the EGFR_P753S mutation [33]. As shown in Figure 1, isoform-specific siRNAs efficiently knocked-down the targeted MEK isoform without affecting the other. We found that individual knock down of either MEK1 or MEK2 had no effect on ERK activation in SK-MEL-28 cells. However, pooled siRNA targeting both MEK1 and MEK2 did cause a decrease in ERK phosphorylation (Figure 1). We also determined the necessity of MEK1 or MEK2 signaling for cell cycle progression in SK-MEL-28 cells using fluorescence-activated cell sorting. Under normal cell culture condition, about 70% of SK-MEL-28 cells are in G_0_/G_1_ phase. Whereas knock down of either MEK1 or MEK2 had no effect on cell cycle progression (*p*>0.05), the combined knockdown of MEK1 and MEK2 resulted in a 10% increase in cells at G_0_/G_1_ (*p*<0.0001) (Table 1). These data indicate that neither MEK1 nor MEK2 signaling alone is necessary for ERK activation and cell cycle progression in SK-MEL-28 cells, and that cells can compensate for loss of either MEK1 or MEK2 to activate ERK and to promote cell cycle progression in SK-MEL-28 cells. This supports the hypothesis that MEK1 and MEK2 are interchangeable for melanoma cell proliferation.

**Table 1:** Percentage of G0/G1 population in MEK siRNA-transfected SK-MEL-28 cells.

siRNA experiments	G_0_/G_1_ population (% ± S.D.)*
None	69.45±0.91
Lipid only	69.68±1.21
Non-silencing	70.49±0.16
MEK1 siRNA-7	70.45±3.27
MEK1 siRNA-8	71.30±3.07
MEK1siRNA-11	70.43±1.83
MEK1 siRNA-7811	72.92±1.71
MEK2 siRNA-9	70.65±0.48
MEK2 siRNA-14	71.21±0.86
MEK2 siRNA-914	71.53±1.24
P1 (MEK1 siRNA-811 + MEK2 siRNA-914)	81.33±1.48 **
P2 (MEK1 siRNA-7811 + MEK2 siRNA-914)	78.24±3.41 **

*Data were obtained from at least three independent experiments.

**p<0.0001, determined by one-way ANOVA followed by Bonferroni post-hoc analysis.

### Point Mutations at the P1′ Position Render MEK Resistant to LF-mediated Cleavage

We reasoned that if MEK1 and MEK2 are functionally redundant and interchangeable, they should have comparable sufficiency for melanoma cell proliferation. For this purpose, we sought to maintain only MEK1 or MEK2 activity while simultaneously deactivating the other MEK isoform as well as other MKK family proteins in melanoma cells. To achieve this, we developed a novel experimental system in which multiple endogenous MEK and MKK pathways were inhibited by LF and MEK1 or MEK2 signaling was rescued by expressing a mutant MEK that was engineered to be resistant to LF-mediated proteolysis.

**Figure 2:**
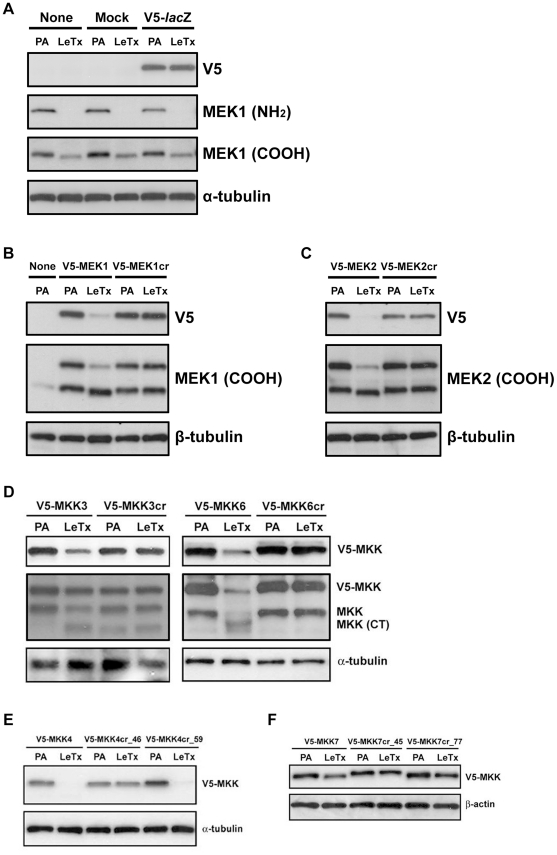
Resistance of V5-MEKcr and V5-MKKcr to LF-mediated proteolysis. (**A**) Non-transfected (None), mock-transfected (Mock) and V5-*lac*Z-transfected CHO K1 cells as well as cells transfected with (**B**) wild-type V5-MEK1 or V5-MEK1cr, (**C**) wild-type V5-MEK2 or V5-MEK2cr, (**D**) wild-type V5-MKK3, V5-MKK3cr, wild-type V5-MKK6, or V5-MKK6cr, (**E**) wild-type V5-MKK4 or V5-MKK4cr, and (**F**) wild-type V5-MKK7 or V5-MKK7cr were treated with PA alone (PA) or LeTx for 12 h as described in Materials and Methods. Total cell lysates were then harvested and immunoblotted with the antibodies indicated to the right of each panel. V5 antibody and an antibody against NH_2_-terminus of MEK1 were used to detect loss of NH_2_-terminal epitopes following LF-mediated proteolysis. Antibodies against the COOH-terminus of MEK1, MEK2, MKK3, and MKK6 were used to detect increased electrophoretic mobility of cleaved MEK/MKK fragments. Antibodies against α-tubulin, β-tubulin, and α-actin were used as equal loading controls.

To make MEK resistant to LF-mediated cleavage, we altered the hydrophobicity and the surface charge of the LF cleavage sites by introducing an aliphatic-to-aspartic acid mutation to the cleavage site at the 1′ position (P1′), which is critical for cleavage by LF [31], [34]. We fused a V5 tag to the NH_2_- termini of wild type and the cleavage resistant form of MEK1 and MEK2 (MEK1cr and MEK2cr), and introduced these V5-MEK fusion proteins into Chinese hamster ovary K1 (CHO K1) cells by transfection. To confirm the LF-mediated cleavage of wild-type V5-MEK and the cleavage resistance of V5-MEKcr, we treated CHO K1 cells expressing V5-MEK or V5-MEKcr with LeTx, and examined the integrity of V5-MEK and V5-MEKcr by immunoblotting. As shown in Figure 2A, in control cells (none-, mock-, and V5-*lac*Z-transfected cells), LeTx treatment resulted in a loss of the NH_2_-terminal epitope (detected by an antibody specifically recognizing MEK1 at the NH_2_- terminus) and a slight increase in the electrophoretic mobility (detected by an antibody recognizing MEK1 at the COOH-terminus) of endogenous MEK1, demonstrating the MEK cleavage by LF in CHO K1 cells. In addition, LF-mediated proteolysis resulted in a loss of NH_2_-terminal V5 epitope only in CHO K1 cells expressing wild-type V5-MEK1 or V5-MEK2 but not in cells expressing V5-MEK1cr or V5-MEK2cr (Figure 2B and 2C). The loss of the V5 epitope did not appear to be a result of decreasing CMV promoter activity (due to the down regulation of c-jun and c-fos by LeTx treatment) as the expression level of V5-*lac*Z in cells remained unchanged in the presence of LF (Figure 2A). We noticed a substantial increase in non-tagged MEK expression accompanied V5-MEK transfection (Figure 2B and C, middle panels). This was due to internal translation from the original start codon of MEK sequence (see Text S1). Together, these results demonstrate that MEK1cr and MEK2cr were resistant to LF-mediated proteolysis.

### Point mutations at the P1′ site render other MKK members resistant to LF-mediated cleavage

A similar strategy was used to generate cleavage-resistant forms of MKK3, MKK4, MKK6 and MKK7. As expected, the aliphatic-to-aspartic mutation at the P1′ position rendered MKK3 and MKK6 resistant to LF cleavage (Figure 2D). As MKK4 and MKK7 were previously reported to harbor two LF cleavage sites [27], two cleavage-resistant mutants with the aliphatic-to-aspartic mutation introduced to one of the cleavage sites were generated for each of MKK4 and MKK7: V5-MKK4cr_46 and V5-MKK4cr_59 for MKK4, and V5-MKK7cr_45 and V5-MKK7cr_77 for MKK7 (Figure 2E and F). Since one of the cleavage sites remained unchanged, we expected that these V5-MKK4cr and V5-MKK7cr mutants should be still sensitive to LF cleavage. However, the aliphatic-to-aspartic mutation was sufficient to make V5-MKK4cr_46 and both of the V5-MKK7cr mutants resistant to cleavage (Figure 2E and F).

### LF cleaves human MKK4 at the Lys^45^-Leu^46^ position but not the Arg^58^-Phe^59^ position in mammalian cells

**Figure 3:**
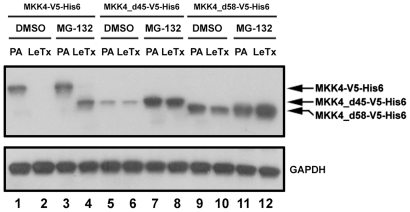
In-cell cleavage of MKK4 by LF. CHO K1 cells were transfected with MKK4-V5-His6, MKK4_d45-V5-His6, or MKK4_d58-V5-His6 plasmids. After transfection, cells were split to four dished and cultured for 12 h. Cells in each dish were treated with PA alone (1 µg/ml PA) or LeTx (1 µg/ml PA plus 0.1 µg/ml LF) in the presence of 0.1% DMSO or 10 µg/ml MG-132 for 24 h. Total cell lysates were collected and immunoblotted with antibodies against V5 epitope (top panel) and GAPDH (bottom panel).

Two possibilities can explain the unexpected observation of MKK4 cleavage. First, LF cleaves MKK4 only at the Lys^45^-Leu^46^ position but not at the Arg^58^-Phe^59^ position (*i.e.*, Arg^58^-Phe^59^ of MKK4 is not a *bona fide* LF cleavage site). Alternatively, LF cleaves MKK4 at both positions in a processive manner (*i.e.*, cleavage at Arg^58^-Phe^59^ requires a prior cleavage at Lys^45^-Leu^46^). To distinguish between these two possibilities we constructed wild-type human MKK4 fused with a V5 tag followed by a 6×His tag at the COOH-terminus (denoted as MKK4-V5-His6). In addition, two MKK4-V5-His6 deletion mutants were constructed as molecular weight indicators: MKK4 with a deletion of amino acid residues 1–45 (denoted as MKK4_d45-V5-His6), and MKK4 with a deletion of amino acid residues 1–58 (denoted as MKK4_d58-V5-His6). The MKK4_d45-V5-His6 deletion mutant also allowed testing of the second possibility that LF cleaves MKK4 at both sites in a processive manner. The set of these proteins was then expressed in CHO K1 cells and in-cell cleavage assays were performed. As shown in Figure 3, LeTx treatment of CHO K1 cells expressing wild-type MKK4-V5-His6 resulted in a complete loss of the V5 epitope, indicating MKK4 cleavage by LF. However, under the same conditions we could not detect the LF-cleaved COOH-terminal fragment of MKK4 using an anti-V5 antibody (Figure 3, lane 2). An explanation for this is that after LF cleavage, the COOH-terminus of MKK4 is degraded. To test this, MG-132, a proteosome inhibitor, was included in the in-cell cleavage assay. As shown in Figure 3 (lane 4), MG-132 treatment rendered the LF-cleaved COOH-terminus of MKK4 recognizable by V5 antibody in the immunoblotting, indicating that after LF cleavage MKK4 is degraded through a proteosome-dependent pathway.

The two MKK4 deletion mutants, MKK4_d45-V5-His6 and MKK4_d58-V5-His6, were distinguishable by the differential electrophoretic mobility in an SDS-PAGE gel (Figure 3, lanes 5–8 and lanes 9–12 respectively). After LF cleavage, the COOH-terminus of MKK4-V5-His6 (Figure 3, lane 4) had a similar electrophoretic mobility as that of MKK4_d45-V5-His6 (Figure 3, lane 5–8) but not MKK4_d58-V5-His6 (Figure 3, lane 9–12). In addition, LeTx did not cause an electrophoretic mobility shift of the MKK4_d45-V5-His6 deletion mutant. It is interesting to note that levels of MKK4_d45-V5-His6 expression were substantially elevated in the presence of MG-132 (Figure 3, lane 7 and 8). This indicates that the MKK4_d45-V5-His5 deletion mutant is rapidly turned over in a proteosome-dependent fashion much like the LeTx-cleaved full length protein. Together these results demonstrate MKK4 is cleaved by LF only at the Lys^45^-Leu^46^ position but not the Arg^58^-Phe^59^ position.

### MKK7 is not a preferred LF substrate in some mammalian cells

**Figure 4:**
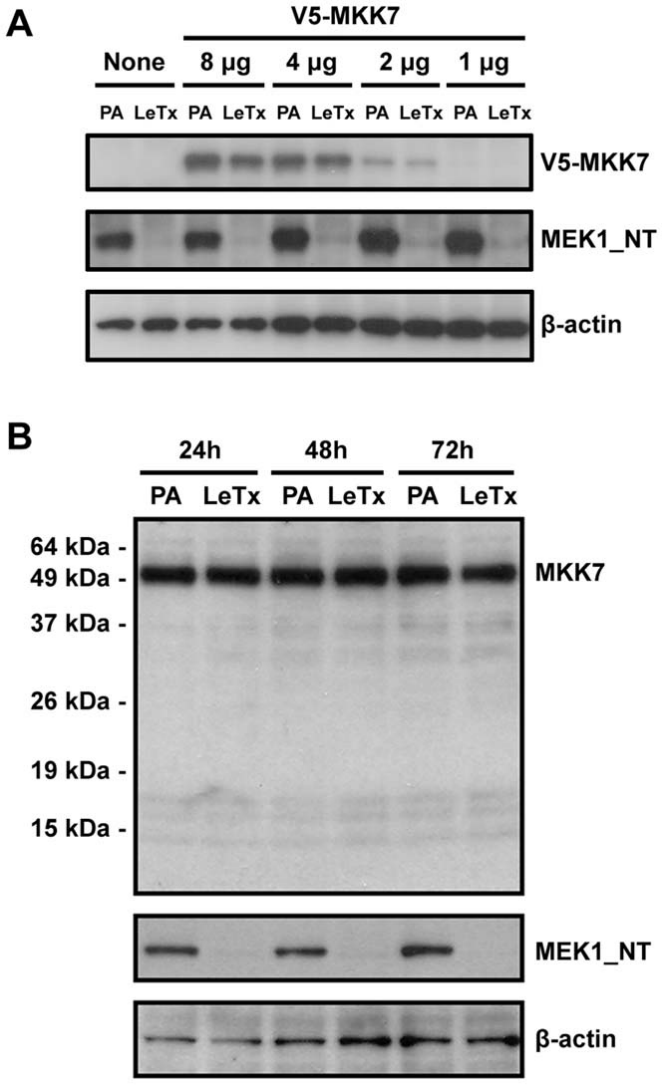
MKK7 is not a preferred LF substrate in mammalian cells. (**A**) Non-transfected (None) CHO K1 cells and cells transfected with a decreasing amount of V5-MKK7 plasmids (8, 4, 2, and 1 µg as indicated) were treated with PA alone (PA) or LeTx for 12 h in the presence of cycloheximide as described in Material and Methods. Total cell lysates were collected and immunoblotted with antibodies against V5 epitope (top panel), NH2-terminus of MEK1 (middle panel), and β-actin (bottom panel). (**B**) To determine whether endogenous MKK7 was cleaved by LF in mammalian cells, 293FT cells were treated with PA alone (PA) or LeTx for 24, 48, and 72 h (indicated). Total cell lysates were collected and immunoblotted with antibodies against the last 20 amino acids of human MKK7 (top panel), NH_2_-terminus of MEK1 (middle panel), and β-actin (bottom panel).

When we tested the cleavage resistance of V5-MKK7cr_45 and V5-MKK7cr_77 mutants, we found both of the mutants were resistant to the cleavage in CHO K1 cells (Figure 2F). However, under the same conditions we were unable to detect convincing cleavage of wild-type MKK7 (Figure 2F). One possible explanation for this is that the amount of LF was insufficient to cleave the excess of V5-MKK7 that was expressed. To test this, a decreasing amount of the plasmid DNA encoding for wild-type V5-MKK7 was transfected into CHO K1 cells and the cleavability of MKK7 was tested in cells in the presence of cycloheximide. Under these conditions, the expression level of wild-type V5-MKK7 was decreased in proportion to the amount of the plasmid DNA transfected (Figure 4A, upper panel). However, LF was unable to cleave wild-type V5- MKK7 even when its expression was decreased to a barely-detectable level. As a positive control for LF activity we probed the same lysates with antibodies against the NH_2_-terminus of MEK1 and observed that MEK1 was cleaved by LF (Figure 4A, middle panel). Moreover, we examined the LF cleavage of endogenous MKK7 in 293FT cells (which are derived from human embryonic kidney 293 cells) using an antibody specifically recognizing the last 20 amino acid of human MKK7. We observed that the endogenous MKK7 in 293FT cells was still intact even when the cells were treated with LeTx for 72 h (Figure 4B). Collectively this result indicates that MKK7 is not a preferred substrate of LF in CHO K1 and 293FT cells.

### Expression and activity of Cleavage-resistant MEK1 or MEK2 in SK-MEL-28 Cells

**Figure 5:**
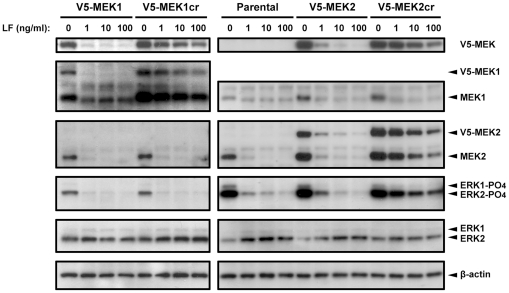
Individual MEK signaling in LeTx-treated SK-MEL-28 cells. SK-MEL-28 parental cells and cells stably expressing V5-MEK or V5-MEKcr were treated with LeTx (1 µg/ml PA plus 0, 1, 10, or 100 ng/ml LF) for 24 h. Total cell lysates were then harvested and immunoblotted with antibodies against the V5 epitope to confirm the cleavage resistance of V5-MEKcr (top panels). Antibodies against the carboxyl terminus of MEK1 (second panel) and the carboxyl terminus of MEK2 (third panel) were used to demonstrate individual MEK expression in LeTx-treated cells. Antibodies against phospho-ERK1/2 (fourth panel) and total ERK1/2 (fifth panel) were used to examine ERK activation, and an antibody against β-actin and β-tubulin were used as a loading control (bottom panels).

To verify cleavage resistance in melanoma cells we established human melanoma SK-MEL-28 cell lines stably expressing V5-MEK1cr or V5-MEK2cr, or the wild-type counterparts. We treated these cells with LeTx, and then examined the cellular levels of MEK1 and MEK2 by immunoblotting. LF-mediated proteolysis caused loss of the NH_2_-terminal V5 epitope in cells expressing wild-type V5-MEK1 and V5-MEK2 within 24 hours even when the cells were treated with low concentrations of LF (1 ng/ml) (Figure 5, top panel). In contrast, V5-MEK1cr and V5-MEK2cr were resistant to LF-mediated cleavage and showed only partial cleavage by LF at the highest concentrations used (100 ng/ml) (Figure 5, top panel).

**Figure 6:**
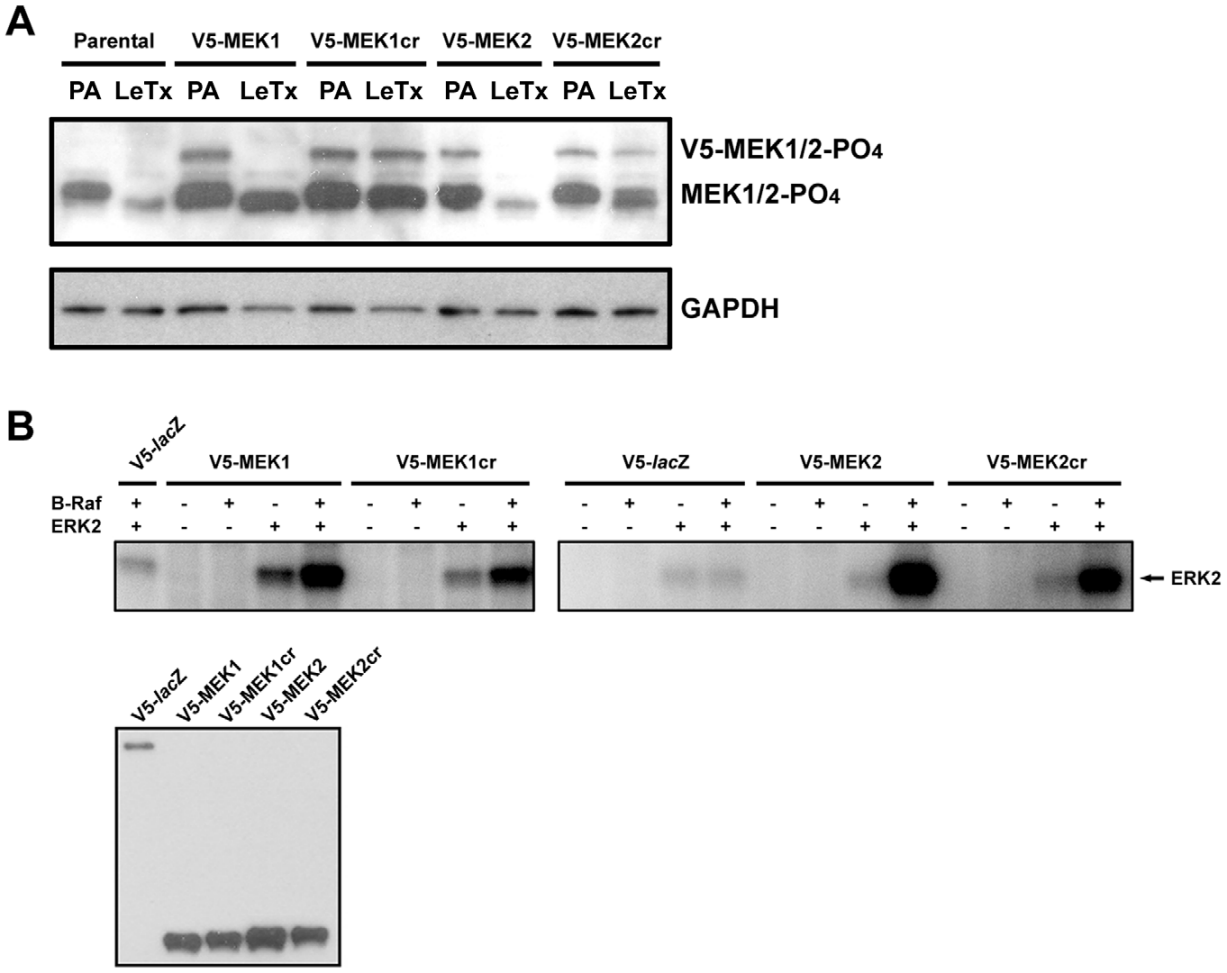
Activity of V5-MEKcr. (**A**) Phosphorylation and activation of V5-MEKcr in cells. Parental SK-MEL-28 cells and cells stably expressing V5-MEK or V5-MEKcr were treated with PA alone (PA) or LeTx for 24 h. Whole cell lysates were then prepared and immunoblotted with anti-phospho MEK1/2 antibody to examine the phosphorylation status of V5-MEK1cr and V5-MEK2cr in SK-MEL-28 cells (upper panel), or anti-GAPDH antibody for equal loading control (lower panel). (**B**) Kinase activity of V5-MEKcr. Anti-V5 immunoprecipitates were prepared from SK-MEL-28 cells and then used for *in vitro* kinase assays (top panels) in the presence or absence of active B-Raf and/or recombinant ERK2. The proteins were then separated in 10% Tris-Glycine gels, and ERK2 phosphorylation was visualized by using a FLA-5000 PhosphoImager. Equal amount of V5 immunoprecipitates were immunoblotted for V5 (bottom panel) as a V5-MEK input control.

Cleavage by LF removes an NH_2_-terminal docking domain that is required for MEK interaction with MAPKs [30], [31], [32]. To confirm that neither the addition of the V5 tag nor the introduction of the LF cleavage-resistant mutation interferes with MEK's ability to interact with and phosphorylate ERK, we examined the activity of V5-MEK1cr and V5-MEK2cr. SK-MEL-28 cells harbor the B-Raf_V600E mutation [1], which promotes constitutive activation of MEK1 and MEK2. When we examined the status of cellular V5-MEK1cr and V5-MEK2cr by immunoblotting using an antibody that only recognized phosphorylated (activated) MEK1 and MEK2, we found that the activation status of V5-MEKcr proteins were comparable to their wild-type counterparts (Figure 6A). This indicates that V5-MEK1cr and V5-MEK2cr are actively signaling in SK-MEL-28 cells. Further, we immunoprecipitated V5-MEK1cr and V5-MEK2cr from SK-MEL-28 cells and then performed *in vitro* kinase assays using recombinant ERK2 as a substrate. Taking into consideration the amount of protein used for each assay, immunoprecipitated V5-MEK1cr and V5-MEK2cr were as capable as their wild-type counterparts of phosphorylating ERK2 (Figure 6B). These results indicate that neither the addition of the V5 tag nor the introduction of the cleavage-resistant mutation alters the ability of MEK to interact with and phosphorylate ERK. Collectively these data demonstrate we can use this model to experimentally isolate either the MEK1 or MEK2 signaling pathway from other MKK signaling pathways in melanoma SK-MEL-28 cells.

### MEK2, but not MEK1, is Sufficient to Maintain ERK Activity

ERK1 and ERK2 are the only identified enzymatic substrates of MEK1 and MEK2. To evaluate the sufficiency of MEK1 and MEK2 to phosphorylate ERK in SK-MEL-28 cells, we examined the status of ERK phosphorylation when these cells had only MEK1 or MEK2 (Figure 5, second and third panels). ERK2 activation was inhibited by LeTx in SK-MEL-28 parental cells as well as in cells expressing wild-type V5-MEK1 or wild-type V5-MEK2 (Figure 5, fourth panel). In contrast, V5-MEK2cr maintained ERK2 phosphorylation in the presence of LeTx. However, ERK2 phosphorylation was not maintained in V5-MEK1cr-expressing cells treated with LeTx. These results indicate that MEK2, but not MEK1, is sufficient to maintain ERK2 activity in the presence of LF, and that MEK1 and MEK2 are not interchangeable for ERK2 activation in this cellular context.

### Genome-wide cDNA Microarray Reveals Non-overlapping Transcriptional Signatures of MEK1 and MEK2

**Figure 7:**
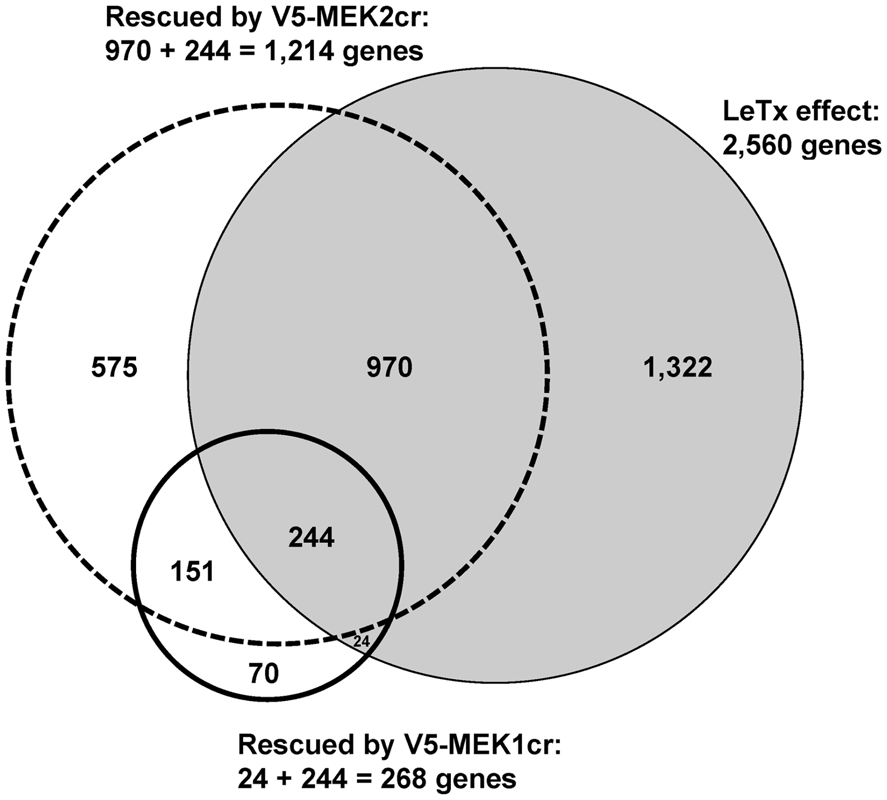
Global gene expression profiles downstream of MEK1 and MEK2. Two sets of SK-MEL-28 parental cells and the cells stably expressing V5-*lac*Z, V5-MEK1, V5-MEK1cr, V5-MEK2, or V5-MEK2cr were treated with control (PA plus LF_E687C) or LeTx for 24 h as described in Material and Methods. Total cell lysates were collected from cells in one of the sets for immunoblotting (see Figure S1). Total RNA samples were collected from the other set of cells and subjected to cDNA microarray hybridization and data analysis as described in Material and Methods. The gene expression profile in control-treated V5-*lac*Z-expressing cells was compared to that in LeTx-treated cells to generate a list of genes that were significantly changed by LeTx treatment (shaded circle). The lists of genes that were significantly rescued by V5-MEK1cr (solid circle) or MEK2 (dashed circle) were generated by comparing the gene expression profile in LeTx-treated V5-*lac*Z-expressing cells with LeTx-treated V5-MEK1cr-expressing cells or LeTx-treated V5-MEK2cr-expressing cells, respectively. The numbers of genes that were significantly changed are labeled.

MAP kinase pathways regulate gene expression at the translational and/or post-translational level. To identify the transcriptional targets of MEK1 and MEK2 we isolated mRNA from MEKcr-expressing SK-MEL-28 cells treated with LeTx. V5-*lac*Z-expressing cells treated with protease inactive LeTx (PA plus LF_E687C, an inactive LF mutant) or active LeTx were used as controls. Under this treatment condition, either MEK1 or MEK2 was expressed in MEKcr-expressing cells (Figure S1). Using the Agilent 60-mer Whole Human Genome Microarrays, we compared the gene expression patterns in these cells. We found that compared with inactive LeTx controls, treatment, of V5-*lacZ*-expressing cells with LeTx resulted in statistically significant changes in expression of 2,560 genes out of 18,359 represented on the microarray. Of these 2,560 genes, 268 can be rescued in V5-MEK1cr-expressing cells, while 1,214 can be rescued in V5-MEK2cr-expressing cells (Figure 7). In examining the transcriptional profiles of each MEK we reasoned that if MEK1 and MEK2 were redundant and interchangeable, transcriptional targets downstream of MEK1 and MEK2 should overlap. In contrast, if MEK1 and MEK2 were not redundant, they should have non-overlapping transcriptional footprints. Consistent with the latter hypothesis we found that the transcriptional footprints of MEK1cr and MEK2cr only partially overlapped. Of note, we found that MEK1cr and MEK2cr affected expression of genes that were not altered by LeTx treatment (see comments in discussion). These observations indicate that in this experimental system MEK1 and MEK2 have both overlapping and non-overlapping transcriptional targets.

### MEK2cr, but not MEK1cr, Rescued Proliferation-related Pathways in LeTx-treated Cells

We further examined the gene expression data to identify pathways that may be differentially regulated by MEK 1 and MEK2 in LeTx-treated cells by performing a gene set enrichment analysis [35], [36]. LeTx treatment resulted in down-regulation of several transcriptional signatures, most of which were proliferation-related pathways, such as pathways of E2F transcription factor, RNA processing, DNA replication and recombination, and cell cycle progression, as well as oncogenic pathways (*e.g.* Myc). This supports the previous findings that LeTx inhibits melanoma cell proliferation *in vitro* [10], [11]. Interestingly, 51 of these transcriptional signatures were uniquely rescued in V5-MEK2cr-expressing cells while no signatures appeared to be uniquely rescued by V5-MEK1cr-expressing cells (Table 2 and Table S1, for Table S1 references please see References S1). This finding indicates that MEK2, but not MEK1, is sufficient for expression of genes associated with cell proliferation.

**Table 2:** Transcriptional signatures that are down regulated by LeTx treatment and significantly rescued by MEK2cr.

	t-statistics^b^			
Signatures/Pathways/Gene sets^a^	V5-*lac*Z	V5-MEK1cr	V5-MEK2cr	Reference^c^
Myc	−7.14	0.32	4.53	Ref. [51]
E2F1, TFDP1, RB1 transcription factors	−6.28	1.07	3.29	MSigDB Gene Set: SGCGSSAAA_V$E2F1DP2_01
rRNA processing	−5.44	0.33	2.74	GO: 0006364
Ribosome biogenesis	−6.46	0.67	3.80	GO: 0042254
DNA recombination	−5.00	1.39	3.30	GO: 0006310
Nucleotidyl-transfer reaction	−3.63	1.39	2.93	Ref. [52]; MSigDB Gene Set: Nucleotidyltransferase_activity
G1/S cell cycle transition	−3.67	1.49	2.77	MSigDB Gene Set: G1PATHWAY

a The complete list of transcriptional signatures is presented in Table S1.

b t-statistics were obtained from three independent microarray experiments, and the scores in MEK1cr-expressing cells have *p* values greater than 0.005.

c MSigDB Gene Sets: http://www.broadinstitute.org/gsea/msigdb/search.jsp; GO (Gene Ontology): http://www.geneontology.org/; Cancer gene modules: http://robotics.stanford.edu/~erans/cancer/browse_by_modules.html.

### MEK2, but not MEK1, is Sufficient for Melanoma Cell Proliferation

**Figure 8:**
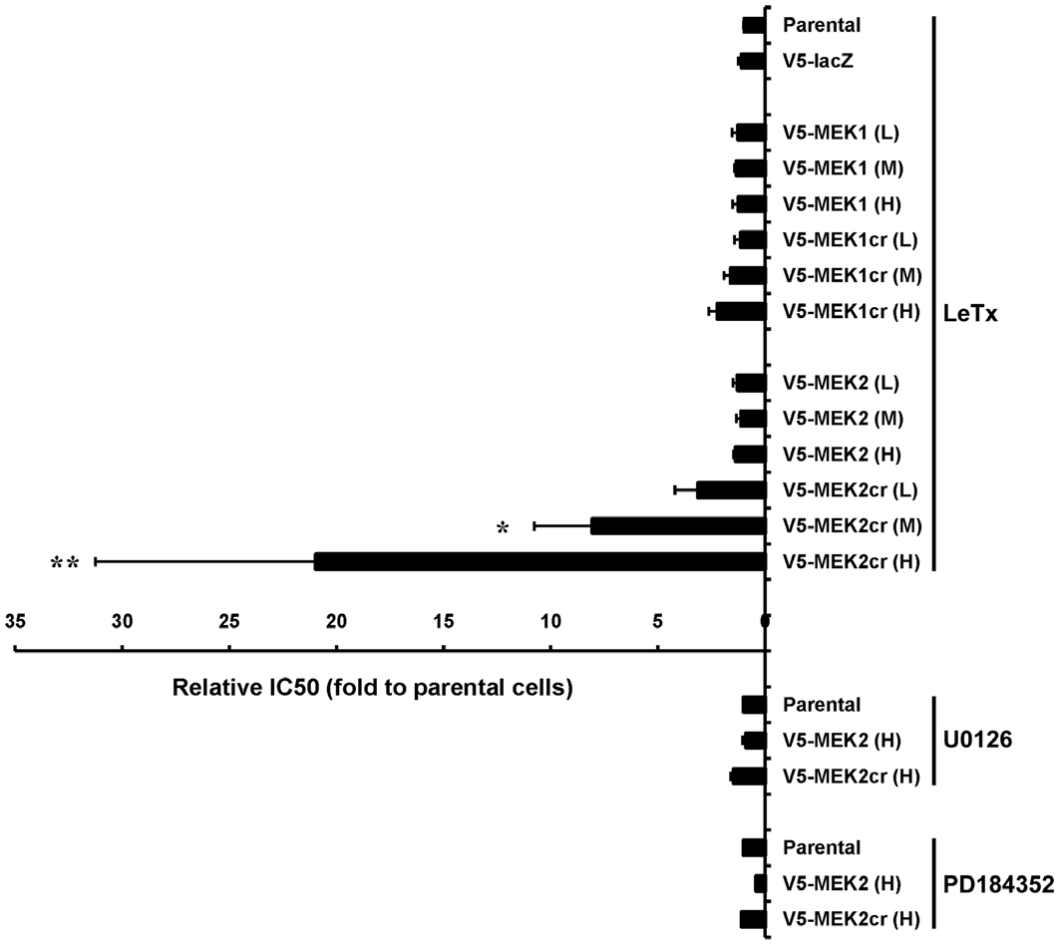
Sensitivity of SK-MEL-28 cells to LeTx and MEK inhibitors. SK-MEL-28 parental cells and the cells stably expressing V5-*lac*Z, low (*L*), moderate (*M*) or high (*H*) levels of wild-type V5-MEK or V5-MEKcr were tested for their sensitivity to LeTx, U0126 and PD184352 by performing *in vitro* proliferation assays as described in Materials and Methods. (A set of representative proliferation curves is presented in Figure S3). IC_50_ values for each stable cell line were normalized to the IC_50_ in parental cells. Results are presented as an average of fold change (*X axis*) of at least three independent experiments ± standard deviation (error bars). Statistical significance of data was performed using one-way ANOVA followed by post-hoc analysis. *P* values were greater than 0.05 except for (*) *p*<0.005 and (**) *p*<0.00001.

Previous studies have shown the inhibitory effect of LeTx on melanoma cell proliferation *in vitro* [10], [11]. To determine whether MEK1 or MEK2 was sufficient for melanoma cell proliferation, we examined the sensitivity of V5-MEKcr-expressing SK-MEL-28 cells for LeTx. To do this, we selected 3 independent stable clones displaying different levels of V5-fusion protein expression from each of the stably transfected SK-MEL-28 cell lines (Figure S2). We tested each of these cell lines for their sensitivity to LeTx *in vitro* by treating the cells with various concentrations of LF (Figure S3). We observed that whereas parental SK-MEL-28 cells and the cells expressing V5-*lac*Z, V5-MEK1, V5-MEK1cr and V5-MEK2 had similar sensitivity to LeTx, cells expressing V5-MEK2cr showed a significantly lower sensitivity to LeTx-induced proliferation inhibition *in vitro* (Figure 8 and Figure S3C). Importantly, the resistance of V5-MEK2cr-expressing cells to LeTx positively correlated with expression levels of V5-MEK2cr. The IC_50_ of LF on SK-MEL-28 cells expressing low, moderate and high levels of V5-MEK2cr was 3, 8, and 21 fold higher than that for parental cells, respectively (Figure 8 and Figure S3C). In addition, we reasoned that if resistance to LF is strictly dependent upon expression of V5-MEK2cr, then these cells should retain their sensitivity to small molecule allosteric inhibitors of MEK. Consistent with this, V5-MEK2cr-expressing cells were as sensitive as other V5-MEK-expressing cells to the MEK inhibitors U0126 and PD184352 (Figure 8). These data demonstrate that the cleavage-resistant form of MEK2, but not MEK1, is sufficient to drive SK-MEL-28 cell proliferation in the presence of LeTx, and that MEK1 and MEK2 signaling are not interchangeable for melanoma cell proliferation.

It was recently reported that cross talk between ERK and p38 MAPK stimulates melanoma proliferation [23]. However, we were unable to detect active p38 MAPK in SK-MEL-28 cells by immunoblotting (data not shown). Furthermore, when we treated SK-MEL-28 cells stably expressing V5-MKK3cr or V5-MKK6cr with LeTx we observed that none of these stable cell lines was resistant to LeTx (Figure S4). These observations indicate that unlike MEK2, MKK3 and MKK6 are not sufficient for melanoma cell proliferation.

### MEK2 is Sufficient for Anchorage-independent Growth

**Figure 9:**
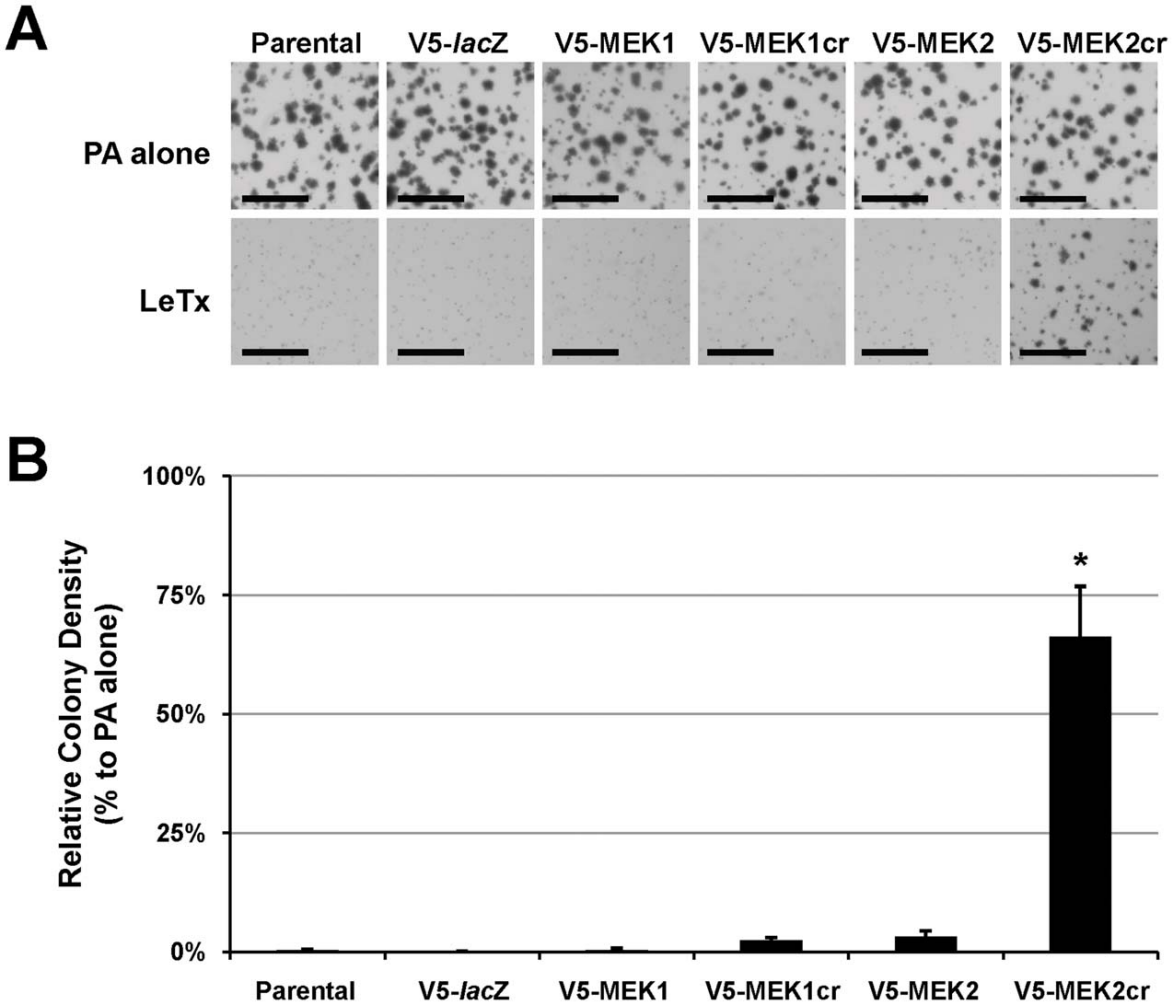
Sufficiency of MEK2 signaling pathway for anchorage-independent growth of SK-MEL-28 cells. SK-MEL-28 parental cells and cells stably expressing V5-*lac*Z or high levels of wild-type V5-MEK or V5-MEKcr were seeded as single-cell suspensions in soft agar and then treated with PA alone control or LeTx, as described in Materials and Methods. After treatment (21 days), colonies were fixed and stained with 1% crystal violet prepared in 10% ethanol. (**A**) Representative images of colonies grown in soft agar in the presence of PA alone or LeTx. Bars, 1 mm. (**B**) Anchorage-independent growth was quantified, and the colony density was determined by dividing the number of colonies by the area and then normalizing to the colony density of the parental cells treated with PA alone. One-way ANOVA followed by post-hoc analysis were performed to determine statistical significance. *P* values were greater than 0.05 except for (*), *p*<0.00001.

We next tried to test the sufficiency of individual MEK1 and MEK2 signaling pathways for tumor growth *in vivo*. However, in xenograft experiments using athymic nude mice we observed that tumors obtained with this model had substantially diminished expression of V5-MEK1cr or V5-MEK2cr (data not shown). This suggests that over-expression of MEK1 or 2 does not provide a selective growth advantage for SK-MEL-28 xenograft tumors in *vivo*. As an alternative approach we evaluated the ability of these cells to grow in an anchorage independent fashion using soft agar colony assays. Parental SK-MEL28 cells and cells expressing V5-*lac*Z, V5-MEK or V5-MEKcr readily formed colonies (>50 µm diameter) within 21 days (Figure 9A, top panels). However, in the presence of LeTx colony formation of all cell types tested with the exception of the cells expressing V5-MEK2cr was inhibited (Figure 9A, lower panels). When these results were quantified we noted that expression of V5-MKK2cr in SK-MEL-28 cells significantly rescued 70% colony formation in the presence of LeTx (Figure 9B). This result demonstrates that MEK2 signaling is sufficient for anchorage-independent growth of SK-MEL-28 cells.

## Discussion

The mitogen-activated protein kinase kinase (MKK) signaling pathways are a group of related protein kinase signaling cascades that are widely expressed in eukaryotes including fungi, plants and animals [37], [38], [39], [40], [41]. For epistemological reasons, MKK signaling pathways have been organized into modules, each of which contains a three tiered kinase cascade comprising a MKK kinase, a MEK or MKK, and a MAPK. This paradigm has shaped our perspective of these pathways so that they are most frequently regarded as distinct. However, it is apparent there is robust cross talk between the MKK signaling pathways (see for example Xia *et al.*, [21], MacKeigan *et al.*, [22], Estrada *et al.*, [23]) and cellular responses to stimuli may result from the coordinated activities of multiple MKK pathways. For this reason we believe it is important to consider potential pathway interactions when evaluating the biologic function of these proteins.

We followed two complementary approaches to test the hypothesis that the functions of MEK1 and MEK2 are critical and interchangeable for SK-MEL-28 melanoma cell proliferation. In the first series of experiments MEK-directed siRNAs were used to selectively knock-down either MEK1 or MEK2, or both, in human melanoma SK-MEL-28 cells to determine the necessity of individual MEK signaling pathway for melanoma cell proliferation. In these experiments we found that simultaneous knockdown of both MEK1 and MEK2 was required to inhibit ERK activation and to block cell cycle progression. This indicates that neither MEK1 nor MEK2 alone is necessary for these activities in melanoma cells and supports our initial hypothesis that MEK1 and MEK2 are interchangeable for melanoma cell proliferation. Other studies support the hypothesis that MEK1 and MEK2 are functionally interchangeable. For example, Scholl *et al.*, show that stepwise deletion of MEK1 or MEK2 alleles reduce Ras-induced epidermal hyperplasia to a similar extent [42]. However, these studies evaluated necessity, but not sufficiency. Cross talk between MKK pathways may mask the potential non-redundancy of MEK1 and MEK2, which may not be revealed until the cross talk is eliminated. As an alternative approach we developed a novel experimental system utilizing the specific proteolytic activity of anthrax lethal toxin to evaluate the sufficiency of individual MEK/MKK signaling pathways for specific phenotypes or cellular functions. This system is applicable to most mammalian cells because anthrax toxin receptors are ubiquitously expressed in many different types of cells [43], [44]. With an appropriate phenotype as a readout (*e.g.* proliferation in this study), this novel system is a powerful tool allowing the dissection of the individual roles of MEK/MKK signaling pathways. Since LF efficiently cleaves and inactivates multiple MKK this experimental approach eliminates not only the possible signaling by residual non-targeted kinase molecules persists, but also ensures there is no cross talk between MKK families.

While developing this model we made two important observations that alter our viewpoint on LF activity. First, we observed MKK4 underwent proteosome-mediated degradation following cleavage by LF. This post-cleavage degradation may not be limited to MKK4 since we noted loss of carboxy-terminal epitope following cleavage for MEK1, MEK2, MKK3, and MKK6 (Figure 2). Based on this we speculate that proteolysis by LF destabilizes MEK/MKKs, perhaps by exposing an aliphatic residue at the NH_2_- terminus [45], rendering them susceptible to proteosomal degradation. Second, we observed in CHO K1 cells that LF cleaves MKK4 only at the Lys^45^-Leu^46^ position but not the Arg^58^-Phe^59^ position, and that LF does not cleave exogenously expressed MKK7 in CHO K1 cells. Similarly, endogenous MKK7 was not cleaved in human 293FT cells. This was unexpected since it was reported earlier that all members in the MKK family with the exception of MEK5 were substrates of LF and that MKK4 and MKK7 each harbor two LF cleavage sites [27]. However, whereas the earlier reports were based on *in vitro* cleavage assays using recombinant protein substrate, our results are based on in-cell cleavage assays. Thus it appears that certain cells provide an environment that restricts LF activity, perhaps by limiting substrate accessibility or by masking the cleavage site, and blocks MKK7 proteolysis.

Interesting and novel observations regarding MEK function also arose from this approach. First, in contrast to our siRNA knockdowns, we found that reconstituting the activity of MEK2, but not MEK1, was sufficient to drive proliferation of SK-MEL-28 cells. This indicates that MEK1 and MEK2 are not functionally redundant. The results obtained from the necessity study (MEK siRNA) and sufficiency study (LeTx and MEKcr) are not actually contradictory as the two experimental systems model fundamentally different cellular contexts. To explain these results, we propose that while MEK2 alone is sufficient for SK-MEL28 melanoma cell proliferation, MEK1 can compensate for loss of MEK2 only in the presence of an as yet unidentified factor. This factor is likely an MKK (but not MEK5) or is regulated by an MKK since the sufficiency of MEK2 is revealed only in the presence of LF, a MEK/MKK-specific protease. However, this factor likely is neither p38 MAPK nor JNK since the basal activities of these kinases in SK-MEL-28 cells are below the limit of detection by immunoblotting. In addition, we do not exclude the possibility that this factor may be an as yet unidentified LF substrate. Identification of this LF-sensitive factor may have important clinical consequences since its targeted inactivation would block the ability of MEK1 to compensate for loss of MEK2 activity and as a consequence increase the dependency on proliferation signaling through MEK2.

The second novel observation arising from this approach was that the transcriptional activities induced by MEK1 and MEK2 were not wholly contained within the LeTx-transcriptional footprint (Figure 7). Since LeTx is a pan MKK inhibitor we expected the result of expression of MEK1cr or MEK2cr would be to relieve a sub-set of LeTx-induced transcriptional changes. However, we found that MEK2cr, and to a lesser extent MEK1cr altered the expression of genes that were unaffected by LeTx. These results may be caused by exogenous expression of MEK2. Alternately, the basal expression levels of these genes may be subject to antagonistic regulation by MEK and at least one of the other MKKs in normal cell culture condition. Changes in expression of these genes are revealed when this co-regulation is imbalanced (when cells have only individual MEK but not other MKKs). There are several examples of MKK-related antagonistic co-regulation in the literature. For example, survival of differentiated rat PC-12 pheochromocytoma cells in culture is dependent upon the presence of nerve growth factor (NGF) and removal of NGF from the medium causes an increase in the activities of p38 MAPK and JNK which is necessary and sufficient to induce apoptosis [21]. Interestingly, a decrease in ERK activity accompanies NGF withdrawal and expression of constitutively activated MEK1 prevents apoptosis induced by NGF withdrawal. These results indicate that apoptosis in NGF-differentiated PC-12 cells is regulated by opposing activities of ERK and p38 MAPK/JNK. Similar results have been obtained for paclitaxel-mediated apoptosis of transformed cells [22].

In summary, using two complementary experimental approaches we have tested the redundancy of MEK1 and MEK2 signaling in the *in vitro* proliferation of SK-MEL-28 melanoma cells. We observed that MEK2, and not MEK1, is sufficient for cell proliferation. However, MEK1 can compensate for the loss of MEK2 activity in the presence of an unidentified factor. Our study provides novel insight into the complicated interplay between MEK1 and MEK2 that may have significant clinical impact for treatment.

## Materials and Methods

### Cell lines and stable cell line establishment

Chinese hamster ovary (CHO) K1 cells were purchased from American Type Culture Collection (Manassas, VA) and grown in Dulbecco's Modified Eagle's Medium (DMEM) supplemented with 10% FBS and 50 units/ml penicillin/streptomycin. SK-MEL-28 cells were obtained from CeeTox Inc. (Kalamazoo, MI) and grown in Roswell Park Memorial Institute (RPMI) −1640 medium supplemented with 5% FBS and 50 units/ml penicillin/streptomycin. 293FT cells were purchased from Invitrogen (Carlsbad, CA) and cultured in DMEM supplemented with 10% FBS and 50 units/ml penicillin/streptomycin. Cells were cultured at 37°C in a humidified 5% CO_2_ incubator. To establish stable cell lines, SK-MEL-28 cells were transfected with V5-MEK, V5-MEKcr or V5-*lac*Z control expression vectors by using Lipofectamine 2000™ (Invitrogen, Carlsbad, CA) according to the manufacturer's instructions. Clonal stable transfectants were selected against Geneticin®. Expression levels of V5-fusion proteins in each stable clone were determined by immunoblotting.

### siRNA-mediated MEK knock down

Multiple siRNAs specifically against human MEK1 or MEK2 and AllStars non-silencing control siRNA were purchased from QIAGEN Inc. (Valencia, CA). To deliver siRNA into SK-MEL-28 cells, we used siLentFect™ Lipid (Bio-Rad, Hercules, CA) as the transfection reagent. To do this, SK-MEL-28 cells (3×10^5^ cells) were seeded in 60-mm dishes. The next day, cells were washed three times with PBS and covered with 1.6 ml of OPTI-MEM®I (Invitrogen, Carlsbad, CA), to which siRNA:Lipid complexes (prepared as described below) were then added. To prepare siRNA:Lipid complexes, 10 µl of lipid was added into 30 µl of OPTI-MEM®I and incubate at room temperature for five minutes. The prepared lipid was then added into 360 µl of OPTI-MEM®I containing 100 pmole of each control or MEK siRNAs plus 100 pmole of AlexaFluor488-conjugated non-silencing siRNA, and incubated at room temperature for 20 minutes to allow siRNA:Lipid complexes form. Eight hours after addition of siRNA:Lipid complexes, cells were then trypsinized and split to two 60-mm dishes and culture in DMEM containing 10% FBS and 50 units/ml penicillin/streptomycin for 72 hours. At this time point, AlexaFluor488 fluorescence could be observed in greater than 95% of transfected cells under a fluorescence microscope (data not shown), indicating that siRNAs were delivered into the cells. Cells were harvested for immunoblotting and cell cycle analysis.

### Cell cycle analysis

Cell cycle profile was assessed by fluorescence-activated cell sorting as previously described [46]. Percentages of cells in G_0_/G_1_ phase of cell cycle were measured using FACSCalibur software program. Statistical analysis of data was performed by one-way ANOVA (www.physics.csbsju.edu/stats/anova_NGROUP_NMAX_form.html) followed by post-hoc analysis (http://graphpad.com/quickcalcs/posttest1.cfm).

### V5-MEKcr and V5-MKKcr constructions

Human MEK1 (NM_002755.2) sequence was PCR-amplified from the pREST-A/MKK1 vector, which was a generous gift from Natalie Ahn [47]. Human MEK2 (NM_030662.2), MKK3 (NM_145109.2), MKK4 (NM_003010.2), and MKK6 (NM_002758.2) sequences were PCR-amplified from the I.M.A.G.E. clones (Open Biosystems, Huntsville, AL): MEK2, clone #2961198; MKK3, clone #5215093; MKK4, clone #5272439; MKK6, clone #4499772. Human MKK7 (NM_145185.2) sequences were PCR-amplified from the pcDNA3/MKK7 vector. Amplified sequences were then inserted into pENTR™ Directional TOPO® vector (Invitrogen, Carlsbad, CA) according to the manufacturer's instructions. To generate MEKcr/MKKcr, aspartic acid residues were introduced into the P1′ position of LF cleavage sites (the 9^th^, 11^th^, 27^th^ and 15^th^ amino acid of MEK1, MEK2, MKK3 and MKK6, respectively; the 46^th^ and 59^th^ amino acids of MKK4; 45^th^ and 77^th^ amino acids of MKK7.) by using QuikChange® Site-Directed Mutagenesis (Agilent, Santa Clara, CA). All the wild-type MEK/MKK and MEKcr/MKKcr sequences were verified by DNA sequencing. V5-MEK/MKK mammalian expression vectors were created by performing LR recombination reactions to move MEK/MKK sequences from the Entry vectors to the pcDNA3.1/nV5-DEST Destination vectors (Invitrogen, Carlsbad, CA) according to the manufacturer's instructions.

### Constructions of MKK4-V5-His6 and the deletion mutants

To make MKK4 carboxyl terminal fusion, the stop codon of MKK4 sequence was removed from the MKK4 Entry vector by using QuikChange® Site-Directed Mutagenesis (Agilent, Santa Clara, CA) with modified PCR reaction in which 10% DMSO was included to solve secondary structure. A second run of modified Site-Directed Mutagenesis reaction was performed to remove amino acid 2–45 and 2–58 of MKK4 from the modified Entry vector in order to generate the deletion mutant MKK4_d45 and MKK4_d58, respectively. The coding regions of MKK4 on all the Entry vectors were verified by DNA sequencing. MKK4-V5-His6 mammalian expression vectors were created by performing LR recombination reactions to move MKK4 sequences from the modified Entry vectors to the pcDNA-DEST40 Destination vectors (Invitrogen, Carlsbad, CA) according to the manufacturer's instructions.

### In-cell MKK/MEK cleavage assay

To confirm the cleavage resistance of MEKcr and MKKcr, we performed cleavage assays in CHO K1 cells. To do this, cells were transfected with V5-MKK/MEK or V5-*lac*Z expression vectors by Lipofectamine™ 2000 (Invitrogen, Carlsbad, CA) according to the manufacturer's instructions. After transfection, cells were trypsinized and split into two dishes, and each was then treated with either control (1 µg/ml PA) or LeTx (1 µg/ml PA and 0.1 mg/ml LF) in the presence of 10 µg/ml cycloheximide (Sigma, St. Louis, MO). PA and LF were expressed in an attenuated strain of Bacillus anthracis (BH445) and purified by fast pressure liquid chromatography as described [48]. Total cell lysates were collected in RIPA lysis buffer [50 mM Tris-HCl pH 7.5, 150 mM NaCl, 1 mM EDTA, 1 mM EGTA, 2 mM Na_3_VO_4_, 20 mM sodium pyrophosphate, 1% sodium deoxycholate, 1% Triton X-100, 0.1% SDS, and 1× EDTA-free protease inhibitor cocktail (Roche, Indianapolis, IN)] and homogenized by sonication. Protein concentrations were determined by BCA™ Protein Assay Kit (Pierce, Rockford, IL) according to the manufacturer's instructions. Lysates were then prepared in 1× SDS sample buffer. Five micrograms of total cell lysates were subjected to immunoblotting to detect LF-mediated cleavage as described in the results.

### LF cleavage of MKK4 and MKK7 in mammalian cells

To determine MKK4 cleavage by LF in mammalian cells, MKK4-V5-His6 and the deletion mutants were transfected into CHO K1 cells by Lipofectamine™ 2000 (Invitrogen, Carlsbad, CA). After transfection, cells were split into four dished and cultured for 12 h. Cells in each dish were treated with PA alone (1 µg/ml PA) or LeTx (1 µg/ml PA plus 0.1 µg/ml LF) in the presence of 0.1% DMSO or 10 µg/ml MG-132 (EMD Chemicals, Gibbstown, NJ) for 24 h. Total cell lysates were collected and subjected to immunoblotting. To determine whether MKK7 was cleaved by LF in mammalian cells, 293FT cells were treated with PA alone (1 µg/ml PA) or LeTx (1 µg/ml PA plus 0.1 µg/ml LF) for 24, 48, and 72 h. Total cell lysates were collected and subjected to immunoblotting.

### *In vitro* kinase assay

To test the kinase activity of MEK and MEKcr, V5-MEK fusion proteins were first immunoprecipitated from SK-MEL-28 stable cell lines as following described. Cells cultured for 24 h were harvested and lysed on ice in lysis buffer (20 mM Tris-HCl pH 7.5, 150 mM NaCl, 1.5 mM MgCl_2_, 2 mM EGTA, 1% Triton X-100, 2 mM DTT, 10 mM NaF, 1 mM Na_3_VO_4_, and 12 mM β-glycerophosphate). Cell lysates were then homogenized by sonication in ice bath, and 200 mg of total lysates were incubated with 25 µl of agarose-immobilized V5 antibody (Bethyl Laboratories, Montgomery, TX) in a total volume of 500 µl of lysis buffer on a rotator in 4°C for 12 h. The precipitates were then washed twice with the lysis buffer and twice with kinase assay buffer (25 mM Tris-HCl pH 7.5, 5 mM β-glycerophosphate, 2 mM DTT, 0.1 mM Na_3_VO_4_, and 10 mM MgCl_2_), and resuspended in 50 µl of kinase assay buffer as the kinase source for the following kinase assay. Five microliters of V5-immunoprecipitate was incubated on ice with or without 0.1 unit of active B-Raf (Upstate Biotechnology, Lake Placid, NY) or 0.1 µg of recombinant ERK2. Assay dilution buffer (20 mM MOPS pH 7.2, 25 mM β-glycerolphosphate, 5 mM EGTA, 1 mM Na_3_VO_4_ and 1 mM DTT) was added to a total volume of 7 µl, and then 3 µl of ATP mixture [0.5 µl of [γ-32P] ATP (10 mCi/ml, 3000 mCi/mmole; Amersham, Piscataway, NJ) in 250 µM ATP and 37.5 mM MgCl_2_] was added. *In vitro* phosphorylation reaction was carried out by incubating the reaction in 30°C for 30 min, and then stopped by addition of 10 µl of 2× SDS sample buffer. The proteins were then separated in 10% Tris-Glycine gels, and ERK2 phosphorylation was visualized by using a FLA-5000 PhosphoImager (Fujifilm Corp., Greenwood, SC). The input of 5 ul of V5-fusion proteins was shown by immunoblotting using V5 antibody.

### Immunoblotting

Total cell lysates were collected in RIPA lysis buffer. Protein concentrations were determined using a BCA™ Protein Assay Kit (Pierce, Rockford, IL). Five micrograms of total cell lysates were separated in 10% Novex® Pre-Cast Tris-Glycine Gels (Invitrogen, Carlsbad, CA) and then electro-transferred onto polyvinylidene fluoride (PVDF) membranes (Millipore, Billerica, MA) according to the manufacturers' instructions. Membranes were then soaked in 5% non-fat milk for 1 hour and hybridized with primary antibodies against V5 epitope (Bethyl Laboratories, 1∶10,000 dilution for CHO K1 cleavage assays and 1∶5,000 dilutions for SK-MEL-28 stable expressions), NH_2_-terminus of MEK1 (Millipore, Billerica, MA, #07-641, 1∶2,000 dilution), COOH-terminus of MEK1 (Santa Cruz Biotechnology, Santa Cruz, CA, #SC-219, 1∶200 dilution), COOH-terminus of MEK2 (Santa Cruz Biotechnology, #SC-525, 1∶200 dilution), COOH-terminus of MKK3 (Santa Cruz Biotechnology, #SC-961, 1∶1,000 dilution), COOH-terminus of MKK6 (Epitomics, Burlingame, CA, #1821-1, 1∶1,000 dilution), COOH-terminus of MKK7 (Epitomics, #1949-1, 1∶1,000 dilution), phosphp-MEK1/2 (Cell Signaling, Danvers, MA, #9154, 1∶1,000 dilution), phospho-ERK (Cell Signaling, #9106, 1∶2,000 dilution), ERK (Cell Signaling, #9102, 1∶1,000 dilution), α-tubulin (Sigma, St. Louis, MO, #T9026, 1∶5,000 dilution), β-tubulin (Sigma, #T5201, 1∶1,000 dilution), β-actin (Sigma, #A1978, 1∶5,000 dilution), or GAPDH (Cell Signaling, #2118, 1∶5,000 dilution). Conditions for primary antibody hybridizations were followed according to the antibody datasheets. After primary antibody hybridization, membranes were washed three times in TBST buffer (50 mM Tris, 150 mM NaCl, and 0.1% Tween-20), hybridized with HRP-conjugated secondary antibodies (Kirkegaard & Perry Laboratories, Gaithersburg, MD) according to the instructions of the antibodies, and then washed three times in TBST buffer. Immunoblotting signals were then detected by LumiGLO™ Reagent and Peroxide (Cell Signaling, Beverly, MA).

### Human cDNA Microarray and transcriptional signature analysis

Two sets of parental SK-MEL-28 cells and cells stably expressing V5-*lac*Z, V5-MEK1, V5-MEK1cr, V5-MEK2, or V5-MEK2cr were treated with LeTx (1 µg/ml PA plus 10 ng/ml LF) or control (1 µg/ml PA plus 10 ng/ml LF_E687C) for 24 h. Total cell lysates were prepared from cells in one of the two sets and subjected to immunoblotting to examine individual MEK1 or MEK2 signaling (see Figure S1). Total RNA samples were prepared from cells in the other set by using *mir*Vana™ RNA isolation kit (Applied Biosystems, Austin, TX). Four RNA samples (control-treated V5-*lac*Z-expressing cells, LeTx-treated V5-*lac*Z-, V5-MEK1cr, and V5-MEK2cr-expressing cells) were submitted to the Microarray Core Laboratory at the Van Andel Research Institute for microarray hybridization. A total of three independent experiments were performed. Gene expression profiles (n = 12) were generated using the Agilent 60-mer Whole Human Genome 44 k Microarray platform according to the manufacturer's instructions. All subsequent analysis was performed using the Bioconductor software environment. Microarray data was processed and normalized using the *limma* Bioconductor package [49], [50]. Microarray data is MIAME compliant and the raw data has been deposited in the GEO database (GEO accession number: GSE23930). Gene set enrichment analysis was then performed using the PGSEA Bioconductor package [35] with only the most significant results shown. A signature was determined to be uniquely rescued by V5-MEK1cr or V5-MEK2cr when it satisfied two criteria. First, the signature in V5-*lac*Z-expressing cells had to have a highly significant (*p*<0.005) positive or negative response to LeTx treatment while in cells expressing V5-MEK1cr or V5-MEK2cr showed a highly significant (*p*<0.005) response to LeTx treatment in the opposite direction. Second, the signature in cells expressing the other V5-MEKcr isoform did not demonstrate a highly significant (*p*<0.005) to LeTx treatment. Differentially expressed genes were identified using a moderated t-statistic as implemented in the *limma* Bioconductor package. Significance values were adjusted using the false discovery rate (FDR) method to compensate for multiple testing.

### *In vitro* proliferation assay

SK-MEL-28 cells (1,500 cells) were cultured in 96-well plates for 24 hours, and then treated with control (1 µg/ml PA) or LeTx (1 µg/ml PA plus 0.01–10,000 ng/ml LF) for 72 hours. For U0126 and PD 184352 treatments, cells were treated with 1–100,000 nM U0126 (EMD Chemicals, Gibbstown, NJ) or 0.01–10,000 nM PD 184352 (USBiological, Marblehead, MA) for 72 h. Cell viability was determined by using CellTiter 96® Aqueous Non-Radioactive Cell Proliferation Assay (Promega, Madison, WI) according to the manufacturer's instructions. LeTx-induced growth inhibition on each stable cell line was presented by plotting the relative viability normalized with PA control treatment against LF concentrations. IC_50_ values were determined by SigmaPlot software (Systat Software, San Jose, CA). Statistical analysis of data was performed by one-way ANOVA (www.physics.csbsju.edu/stats/anova_NGROUP_NMAX_form.html) followed by post-hoc analysis (http://graphpad.com/quickcalcs/posttest1.cfm).

### Anchorage-independent growth assay

Anchorage-independent growth assays were performed as described [46]. Briefly, 10,000 cells were prepared as single cell suspensions in 0.35% agar prepared in a volume of 1 ml of culture media, and then seeded onto 1 ml of hard agar (1% agar prepared in culture media) in 12-well plate. One day after cell seeding, 1 ml of culture media containing PA along control (1 µg/ml PA) or LeTx (1 µg/ml PA and 0.01 µg/ml LF) were added on the top of cell-containing agar layer. Cells were then incubated at 37°C in a humidified 5% CO_2_ incubator. The PA- or LeTx-containing culture media was replaced every 2–3 days for a total of 21 days. Colonies were then fixed and stained with 1% crystal violet in 10% ethanol for observation. Images were then taken under a dissecting microscope. Colonies >50 µm in diameter were quantified using Imagine software as previously described [46]. Colony density was normalized to control untreated samples.

## Supporting Information

Figure S1 **Individual MEK1 or MEK2 in SK-MEL-28 cells.** Two sets of SK-MEL-28 parental cells and the cells stably expressing V5-*lac*Z, V5-MEK1, V5-MEK1cr, V5-MEK2, or V5-MEK2cr were treated with PA plus LF_E687C control (EC) or LeTx (LF) for 24 h as described in Material and Methods. Total RNA samples were collected from one of the two sets of cells, and subjected to cDNA microarray hybridization and data analysis. Total cell lysates were collected from cells in the other set for immunoblotting probed with antibodies against V5 epitope (top panel), NH_2_-terminus of MEK1 (the second panel), NH_2_-terminus of MEK2 (the third panel), phospho-ERK1/2 (the fourth panel), total ERK1/2 (the fifth panel), and GAPDH (bottom panel).
(TIF)

Figure S2 **Expression levels of V5 fusion proteins in SK-MEL-28 cells.** SK-MEL-28 cells stably expressing V5-*lac*Z, wild-type V5-MEK or V5-MEKcr were established as described. Total cell lysates were harvested and subjected for immunoblotting by using antibody against V5 epitope to detect expression levels of V5 fusion proteins (upper panel) and antibody against α-tubulin for equal loading control (lower panel). Parental SK-MEL-28 cells (P) and cells stably expressing V5-*lac*Z (Z) were used as controls. High (H), moderate (M) and low (L) expression levels of V5-MEK are indicated.
(TIF)

Figure S3 **Sensitive of SK-MEL-28 stable cells to LeTx** ***in vitro*** **.** SK-MEL-28 parental cells (**A**, solid line) and the cells stably expressing V5-*lac*Z (**A**, dashed line), V5-MEK1 (**B**, green lines), V5-MEK1cr (**B**, red lines), V5-MEK2 (**C**, green lines) or V5-MEK2cr (**C**, red lines) with different V5-fusion protein expression levels: low (**B** and **C**, dashed lines), moderate (**B** and **C**, thin solid lines) or high (**B** and **C**, thick solid lines) were tested for the sensitive to LeTx by doing a *in vitro* proliferation assay in the presence of LeTx as described. The *x*-axis represents relative viability normalized by PA alone-treated control. The *y*-axis represents the concentration of LF. Data presented in this figure is a representative of three independent experiments. Error bars represent standard divisions of triplicate wells in the assay.
(TIF)

Figure S4 **Sensitivity of SK-MEL-28 stable cell stably expressing V5-MKK3cr or V5-MKK6cr to LeTx** ***in vitro*** **.** SK-MEL-28 parental cells, cells stably expressing V5-*lac*Z, and cells stably expressing low (L), moderate (M), or high (H) levels of V5-*lac*Z, wild-type V5-MKK, or V5-MKKcr (indicated) were tested for their sensitivity to LeTx as described in Materials and Methods. Results are presented as described in the legend of Figure 8. One-way ANOVA followed by post-hoc analysis on all the 26 clones (presented in Figure 8 and here) showed that only the V5-MEK2cr(M), V5-MEK2cr(H), and V5-MKK6cr(L) have statistically higher IC_50_ values compared to the parental line, with *p* values less than 0.003, 0.00001, and 0.05, respectively (the *p* value for the V5-MKK6(H) clone was >0.05). However, we are reluctant to conclude that expression of V5-MKK6cr protects SK-MEL-28 cells from the effect of LeTx because the increased resistance was not observed in other clones expressing moderate or high V5-MKK6cr levels.
(TIF)

Table S1 **Transcriptional signatures that are down-regulated by LeTx treatment and significantly rescued by MEK2cr.** *^a^* t-statistics were obtained from three independent microarray experiments, and the scores in MEK1cr-expressing cells have *p* values greater than 0.005. *^b^* MSigDB Gene Sets: http://www.broadinstitute.org/gsea/msigdb/search.jsp; GO (Gene Ontology): http://www.geneontology.org/; Cancer gene modules: http://robotics.stanford.edu/~erans/cancer/browse_by_modules.html
(DOCX)

Text S1 Supplemental note
(DOCX)

References S1 Supplemental References
(DOCX)

## References

[1] Davies H, Bignell GR, Cox C, Stephens P, Edkins S (2002). Mutations of the BRAF gene in human cancer.. Nature.

[2] Tsao H, Zhang X, Fowlkes K, Haluska FG (2000). Relative Reciprocity of NRAS and PTEN/MMAC1 Alterations in Cutaneous Melanoma Cell Lines.. Cancer Res.

[3] Cohen C, Zavala-Pompa A, Sequeira JH, Shoji M, Sexton DG (2002). Mitogen-actived Protein Kinase Activation Is an Early Event in Melanoma Progression.. Clin Cancer Res.

[4] Govindarajan B, Bai X, Cohen C, Zhong H, Kilroy S (2003). Malignant Transformation of Melanocytes to Melanoma by Constitutive Activation of Mitogen-activated Protein Kinase Kinase (MAPKK) Signaling.. J Biol Chem.

[5] Tanami H, Imoto I, Hirasawa A, Yuki Y, Sonoda I (2004). Involvement of overexpressed wild-type BRAF in the growth of malignant melanoma cell lines.. Oncogene.

[6] Wellbrock C, Ogilvie L, Hedley D, Karasarides M, Martin J (2004). V599EB-RAF is an Oncogene in Melanocytes.. Cancer Res.

[7] Trisciuoglio D, Iervolino A, Zupi G, Del Bufalo D (2005). Involvement of PI3K and MAPK Signaling in bcl-2-induced Vascular Endothelial Growth Factor Expression in Melanoma Cells.. Mol Biol Cell.

[8] Collisson EA, De A, Suzuki H, Gambhir SS, Kolodney MS (2003). Treatment of Metastatic Melanoma with an Orally Available Inhibitor of the Ras-Raf-MAPK Cascade.. Cancer Res.

[9] Solit DB, Garraway LA, Pratilas CA, Sawai A, Getz G (2006). BRAF mutation predicts sensitivity to MEK inhibition.. Nature.

[10] Koo H-M, VanBrocklin M, McWilliams MJ, Leppla SH, Duesbery NS (2002). Apoptosis and melanogenesis in human melanoma cells induced by anthrax lethal factor inactivation of mitogen-activated protein kinase kinase.. Proceedings of the National Academy of Sciences of the United States of America.

[11] Abi-Habib RJ, Urieto JO, Liu S, Leppla SH, Duesbery NS (2005). BRAF status and mitogen-activated protein/extracellular signal-regulated kinase kinase 1/2 activity indicate sensitivity of melanoma cells to anthrax lethal toxin.. Mol Cancer Ther.

[12] Roberts PJ, Der CJ (2007). Targeting the Raf-MEK-ERK mitogen-activated protein kinase cascade for the treatment of cancer.. Oncogene.

[13] Lee CS, Duesbery NS (2010). Highly Selective MEK inhibitors.. Current Enzyme Inhibition.

[14] LoRusso PM, Adjei AA, Varterasian M, Gadgeel S, Reid J (2005). Phase I and Pharmacodynamic Study of the Oral MEK Inhibitor CI-1040 in Patients With Advanced Malignancies.. J Clin Oncol.

[15] Eisen T, Ahmad T, Flaherty KT, Gore M, Kaye S (2006). Sorafenib in advanced melanoma: a Phase II randomised discontinuation trial analysis.. Br J Cancer.

[16] Scholl FA, Dumesic PA, Barragan DI, Harada K, Bissonauth V (2007). Mek1/2 MAPK kinases are essential for Mammalian development, homeostasis, and Raf-induced hyperplasia.. Dev Cell.

[17] Giroux S, Tremblay M, Bernard D, Cardin-Girard JF, Aubry S (1999). Embryonic death of Mek1-deficient mice reveals a role for this kinase in angiogenesis in the labyrinthine region of the placenta.. Current Biology.

[18] Belanger L-F, Roy S, Tremblay M, Brott B, Steff A-M (2003). Mek2 Is Dispensable for Mouse Growth and Development.. Mol Cell Biol.

[19] Voisin L, Julien C, Duhamel S, Gopalbhai K, Claveau I (2008). Activation of MEK1 or MEK2 isoform is sufficient to fully transform intestinal epithelial cells and induce the formation of metastatic tumors.. BMC Cancer.

[20] Scholl FA, Dumesic PA, Barragan DI, Harada K, Charron J (2009). Selective Role for Mek1 but not Mek2 in the Induction of Epidermal Neoplasia.. Cancer Res.

[21] Xia Z, Dickens M, Raingeaud J, Davis RJ, Greenberg ME (1995). Opposing effects of ERK and JNK-p38 MAP kinases on apoptosis.. Science.

[22] MacKeigan JP, Collins TS, Ting JP (2000). MEK inhibition enhances paclitaxel-induced tumor apoptosis.. J Biol Chem.

[23] Estrada Y, Dong J, Ossowski L (2009). Positive crosstalk between ERK and p38 in melanoma stimulates migration and in vivo proliferation.. Pigment Cell Melanoma Res.

[24] Duesbery NS, Webb CP, Leppla SH, Gordon VM, Klimpel KR (1998). Proteolytic Inactivation of MAP-Kinase-Kinase by Anthrax Lethal Factor.. Science.

[25] Singh Y, Liang X, Duesbery NS, Proft T (2005). Pathogenesis of Bacillus anthracis: the role of Anthrax toxins.. Microbial Toxins Molecular and Cellular Biology.

[26] Pellizzari R, Guidi-Rontani C, Vitale G, Mock M, Montecucco C (1999). Anthrax lethal factor cleaves MKK3 in macrophages and inhibits the LPS/IFNgamma-induced release of NO and TNFalpha.. FEBS Lett.

[27] Vitale G, Bernardi L, Napolitani G, Mock M, Montecucco C (2000). Susceptibility of mitogen-activated protein kinase kinase family members to proteolysis by anthrax lethal factor.. Biochem J.

[28] Vitale G, Pellizzari R, Recchi C, Napolitani G, Mock M (1998). Anthrax Lethal Factor Cleaves the N-Terminus of MAPKKs and Induces Tyrosine/Threonine Phosphorylation of MAPKs in Cultured Macrophages.. Biochemical and Biophysical Research Communications.

[29] Duesbery NS, Resau J, Webb CP, Koochekpour S, Koo HM (2001). Suppression of ras-mediated transformation and inhibition of tumor growth and angiogenesis by anthrax lethal factor, a proteolytic inhibitor of multiple MEK pathways.. Proceedings of the National Academy of Sciences of the United States of America.

[30] Tanoue T, Adachi M, Moriguchi T, Nishida E (2000). A conserved docking motif in MAP kinases common to substrates, activators and regulators.. Nat Cell Biol.

[31] Chopra AP, Boone SA, Liang X, Duesbery NS (2003). Anthrax Lethal Factor Proteolysis and Inactivation of MAPK Kinase.. J Biol Chem.

[32] Bardwell AJ, Abdollahi M, Bardwell L (2004). Anthrax lethal factor-cleavage products of MAPK (mitogen-activated protein kinase) kinases exhibit reduced binding to their cognate MAPKs.. Biochem J.

[33] Ikediobi ON, Davies H, Bignell G, Edkins S, Stevens C (2006). Mutation analysis of 24 known cancer genes in the NCI-60 cell line set.. Mol Cancer Ther.

[34] Park JM, Greten FR, Li Z-W, Karin M (2002). Macrophage Apoptosis by Anthrax Lethal Factor Through p38 MAP Kinase Inhibition.. Science.

[35] Furge KA, Chen J, Koeman J, Swiatek P, Dykema K (2007). Detection of DNA copy number changes and oncogenic signaling abnormalities from gene expression data reveals MYC activation in high-grade papillary renal cell carcinoma.. Cancer Res.

[36] Furge KA, Tan MH, Dykema K, Kort E, Stadler W (2007). Identification of deregulated oncogenic pathways in renal cell carcinoma: an integrated oncogenomic approach based on gene expression profiling.. Oncogene.

[37] Davis RJ (2000). Signal transduction by the JNK group of MAP kinases.. Cell.

[38] Roux PP, Blenis J (2004). ERK and p38 MAPK-activated protein kinases: a family of protein kinases with diverse biological functions.. Microbiol Mol Biol Rev.

[39] Chen Z, Gibson TB, Robinson F, Silvestro L, Pearson G (2001). MAP kinases.. Chem Rev.

[40] Johnson GL, Lapadat R (2002). Mitogen-activated protein kinase pathways mediated by ERK, JNK, and p38 protein kinases.. Science.

[41] Chang L, Karin M (2001). Mammalian MAP kinase signalling cascades.. Nature.

[42] Scholl FA, Dumesic PA, Barragan DI, Charron J, Khavari PA (2009). Mek1/2 gene dosage determines tissue response to oncogenic Ras signaling in the skin.. Oncogene.

[43] Bradley KA, Mogridge J, Mourez M, Collier RJ, Young JA (2001). Identification of the cellular receptor for anthrax toxin.. Nature.

[44] Scobie HM, Rainey GJ, Bradley KA, Young JA (2003). Human capillary morphogenesis protein 2 functions as an anthrax toxin receptor.. Proc Natl Acad Sci U S A.

[45] Tasaki T, Kwon YT (2007). The mammalian N-end rule pathway: new insights into its components and physiological roles.. Trends Biochem Sci.

[46] Ding Y, Boguslawski EA, Berghuis BD, Young JJ, Zhang Z (2008). Mitogen-activated protein kinase kinase signaling promotes growth and vascularization of fibrosarcoma.. Mol Cancer Ther.

[47] Mansour SJ, Resing KA, Candi JM, Hermann AS, Gloor JW (1994). Mitogen-activated protein (MAP) kinase phosphorylation of MAP kinase kinase: determination of phosphorylation sites by mass spectrometry and site-directed mutagenesis.. J Biochem.

[48] Bromberg-White JL, Duesbery NS (2008). Biological and biochemical characterization of anthrax lethal factor, a proteolytic inhibitor of MEK signaling pathways.. Methods Enzymol.

[49] Gentleman RC, Carey VJ, Bates DM, Bolstad B, Dettling M (2004). Bioconductor: open software development for computational biology and bioinformatics.. Genome Biol.

[50] Patterson TA, Lobenhofer EK, Fulmer-Smentek SB, Collins PJ, Chu TM (2006). Performance comparison of one-color and two-color platforms within the MicroArray Quality Control (MAQC) project.. Nat Biotechnol.

[51] Bild AH, Yao G, Chang JT, Wang Q, Potti A (2006). Oncogenic pathway signatures in human cancers as a guide to targeted therapies.. Nature.

[52] Ashburner M, Ball CA, Blake JA, Botstein D, Butler H (2000). Gene ontology: tool for the unification of biology. The Gene Ontology Consortium.. Nat Genet.

