# Supplementary figures and images for: MEK2 Is Sufficient but Not Necessary for Proliferation and Anchorage-Independent Growth of SK-MEL-28 Melanoma Cells
Source: PLoS One. 2011 Feb 18;6(2):e17165. doi: 10.1371/journal.pone.0017165 (PMC3041822; doi:10.1371/journal.pone.0017165)

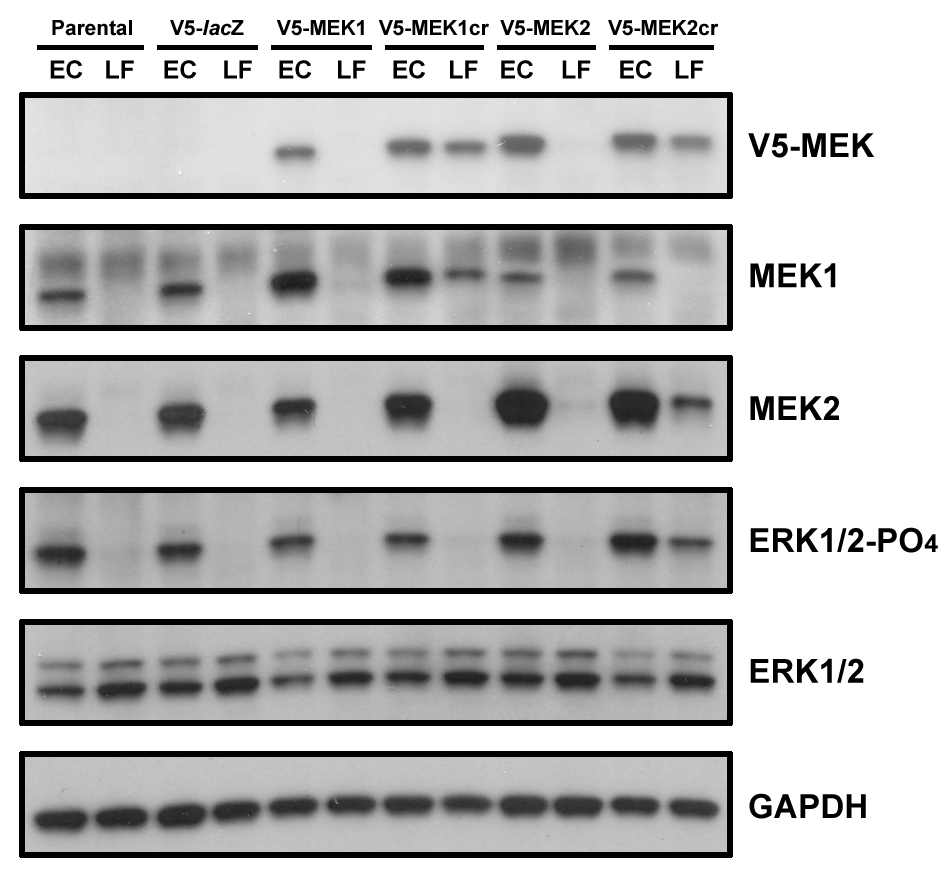

Supplement: Figure S1 — Individual MEK1 or MEK2 in SK-MEL-28 cells. Two sets of SK-MEL-28 parental cells and the cells stably expressing V5-lacZ, V5-MEK1, V5-MEK1cr, V5-MEK2, or V5-MEK2cr were treated with PA plus LF_E687C control (EC) or LeTx (LF) for 24 h as described in Material and Methods. Total RNA samples were collected from one of the two sets of cells, and subjected to cDNA microarray hybridization and data analysis. Total cell lysates were collected from cells in the other set for immunoblotting probed with antibodies against V5 epitope (top panel), NH2-terminus of MEK1 (the second panel), NH2-terminus of MEK2 (the third panel), phospho-ERK1/2 (the fourth panel), total ERK1/2 (the fifth panel), and GAPDH (bottom panel). (TIF) [file pone.0017165.s001.tif]

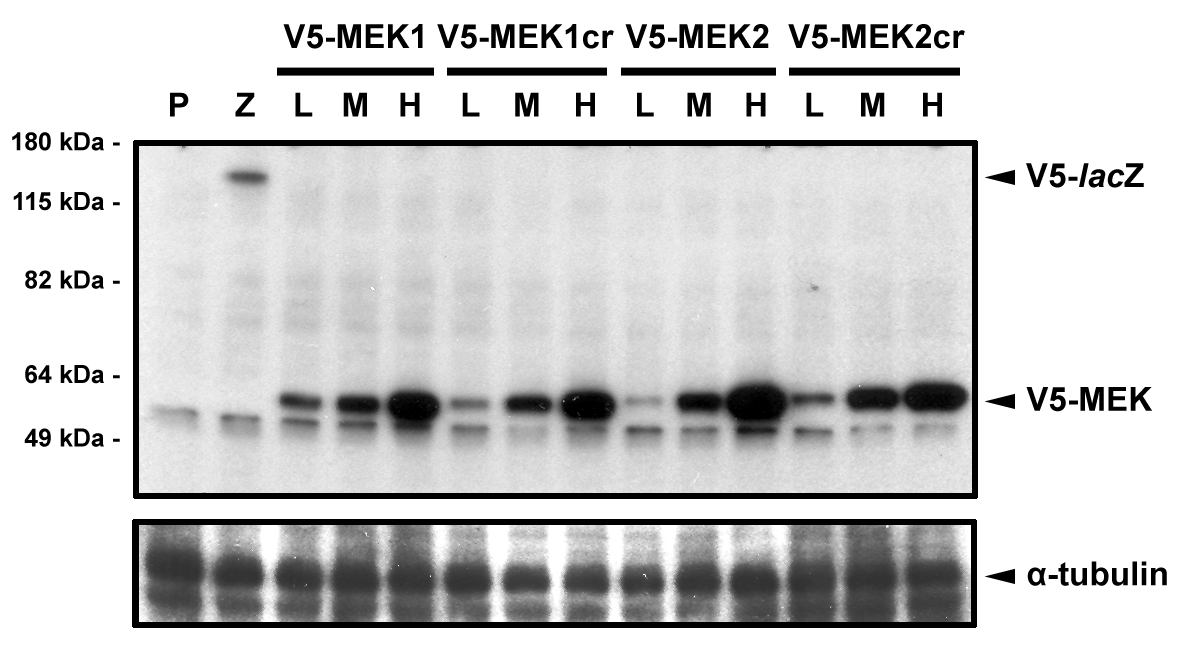

Supplement: Figure S2 — Expression levels of V5 fusion proteins in SK-MEL-28 cells. SK-MEL-28 cells stably expressing V5-lacZ, wild-type V5-MEK or V5-MEKcr were established as described. Total cell lysates were harvested and subjected for immunoblotting by using antibody against V5 epitope to detect expression levels of V5 fusion proteins (upper panel) and antibody against α-tubulin for equal loading control (lower panel). Parental SK-MEL-28 cells (P) and cells stably expressing V5-lacZ (Z) were used as controls. High (H), moderate (M) and low (L) expression levels of V5-MEK are indicated. (TIF) [file pone.0017165.s002.tif]

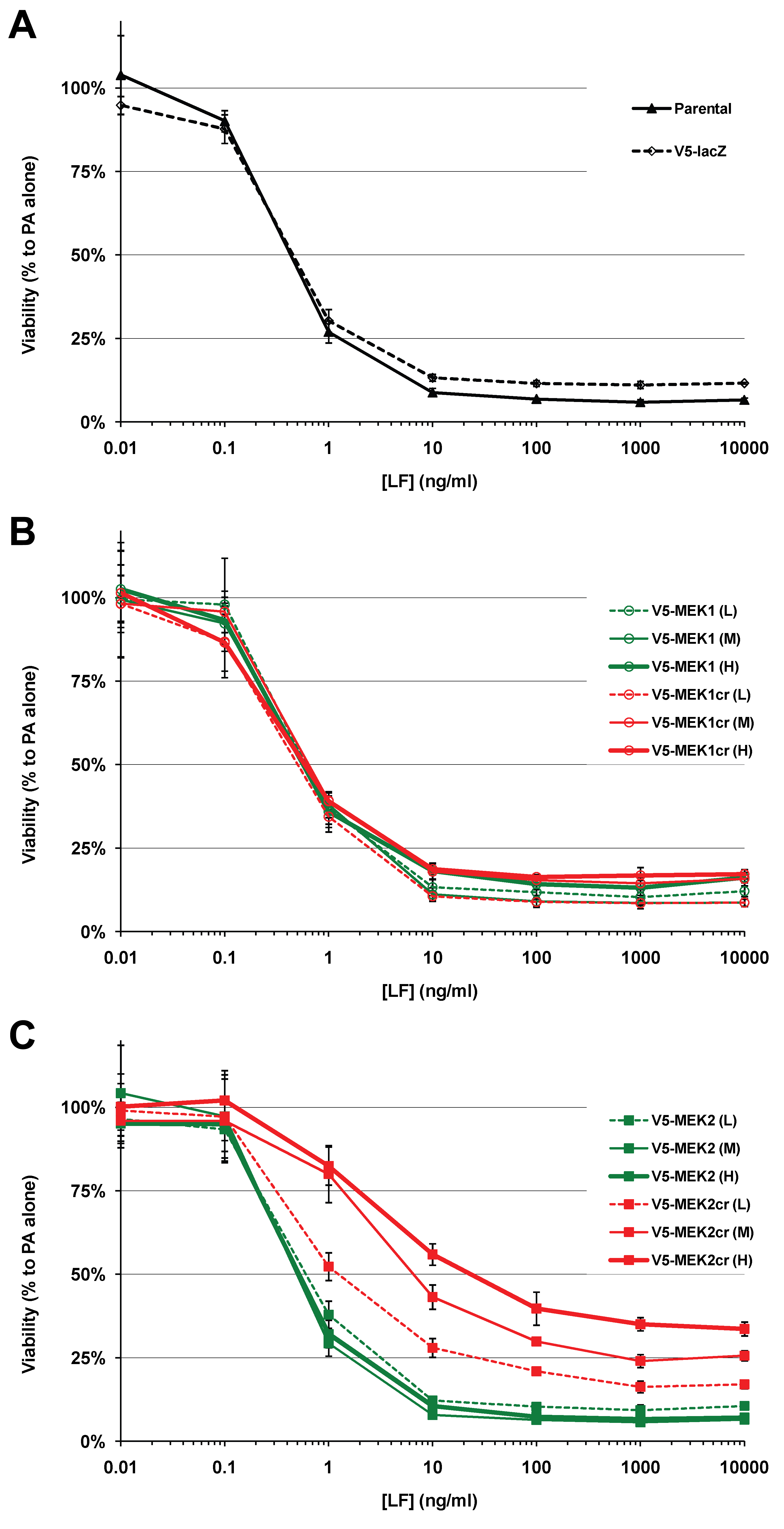

Supplement: Figure S3 — Sensitive of SK-MEL-28 stable cells to LeTx in vitro . SK-MEL-28 parental cells (A, solid line) and the cells stably expressing V5-lacZ (A, dashed line), V5-MEK1 (B, green lines), V5-MEK1cr (B, red lines), V5-MEK2 (C, green lines) or V5-MEK2cr (C, red lines) with different V5-fusion protein expression levels: low (B and C, dashed lines), moderate (B and C, thin solid lines) or high (B and C, thick solid lines) were tested for the sensitive to LeTx by doing a in vitro proliferation assay in the presence of LeTx as described. The x-axis represents relative viability normalized by PA alone-treated control. The y-axis represents the concentration of LF. Data presented in this figure is a representative of three independent experiments. Error bars represent standard divisions of triplicate wells in the assay. (TIF) [file pone.0017165.s003.tif]

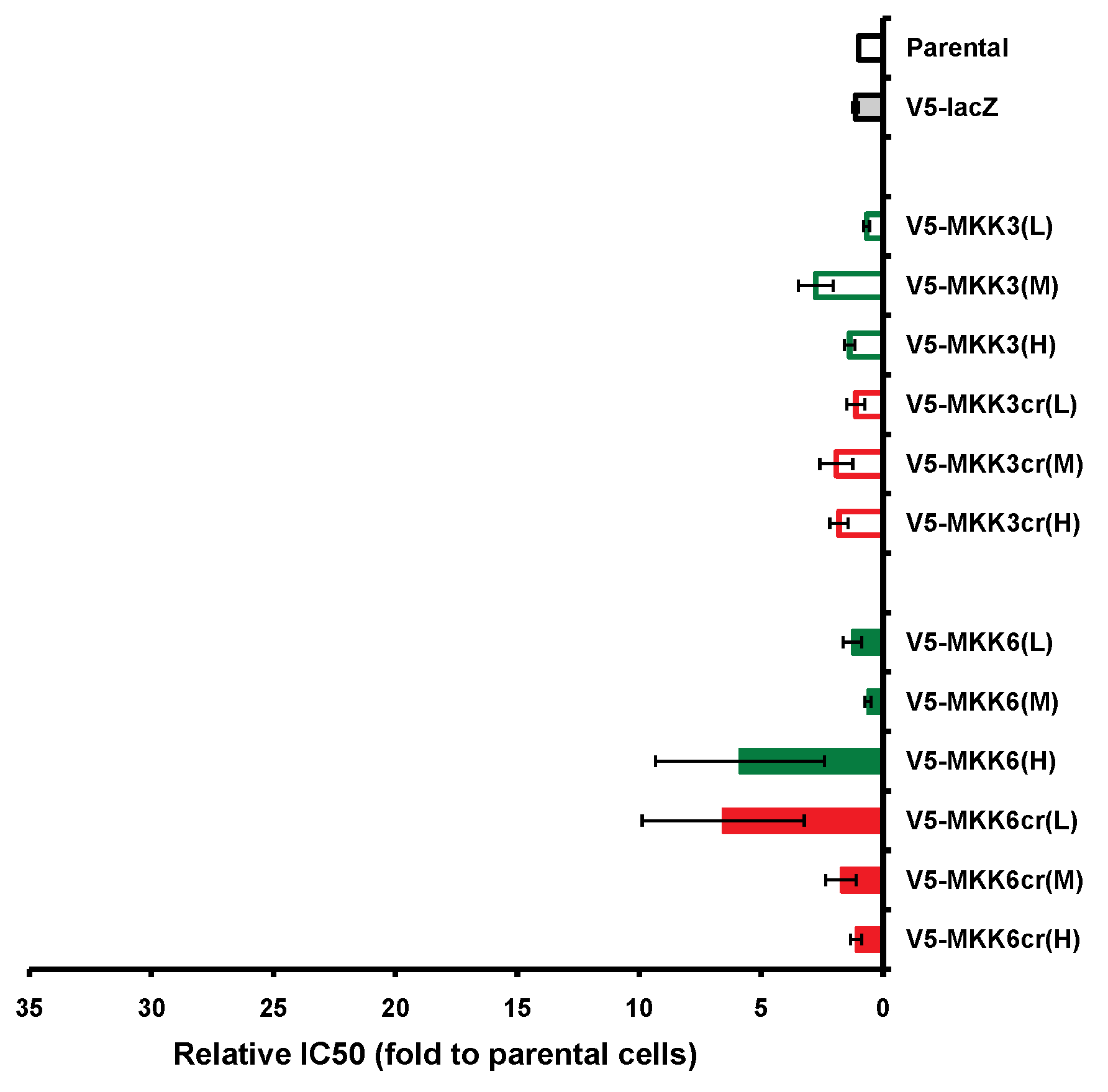

Supplement: Figure S4 — Sensitivity of SK-MEL-28 stable cell stably expressing V5-MKK3cr or V5-MKK6cr to LeTx in vitro . SK-MEL-28 parental cells, cells stably expressing V5-lacZ, and cells stably expressing low (L), moderate (M), or high (H) levels of V5-lacZ, wild-type V5-MKK, or V5-MKKcr (indicated) were tested for their sensitivity to LeTx as described in Materials and Methods. Results are presented as described in the legend of Figure 8. One-way ANOVA followed by post-hoc analysis on all the 26 clones (presented in Figure 8 and here) showed that only the V5-MEK2cr(M), V5-MEK2cr(H), and V5-MKK6cr(L) have statistically higher IC50 values compared to the parental line, with p values less than 0.003, 0.00001, and 0.05, respectively (the p value for the V5-MKK6(H) clone was >0.05). However, we are reluctant to conclude that expression of V5-MKK6cr protects SK-MEL-28 cells from the effect of LeTx because the increased resistance was not observed in other clones expressing moderate or high V5-MKK6cr levels. (TIF) [file pone.0017165.s004.tif]
