# Supplementary material for: MEK2 Is Sufficient but Not Necessary for Proliferation and Anchorage-Independent Growth of SK-MEL-28 Melanoma Cells
Source: PLoS One. 2011 Feb 18;6(2):e17165. doi: 10.1371/journal.pone.0017165 (PMC3041822; doi:10.1371/journal.pone.0017165)
Supplement: Table S1 — Transcriptional signatures that are down-regulated by LeTx treatment and significantly rescued by MEK2cr. a t-statistics were obtained from three independent microarray experiments, and the scores in MEK1cr-expressing cells have p values greater than 0.005. b MSigDB Gene Sets: http://www.broadinstitute.org/gsea/msigdb/search.jsp; GO (Gene Ontology): http://www.geneontology.org/; Cancer gene modules: http://robotics.stanford.edu/~erans/cancer/browse_by_modules.html (DOCX) [file pone.0017165.s005.docx]

**Table S1. Transcriptional signatures that are down-regulated by LeTx treatment and significantly rescued by MEK2cr**

|  | t-statistics *^a^* | | |  |
| --- | --- | --- | --- | --- |
| Signatures/Pathways/Gene sets | V5-*lac*Z | V5-MEK1cr | V5-MEK2cr | Reference *^b^* |
| Myc | -7.14 | 0.32 | 4.53 | Ref. [1] |
| Myc and Myc-associated factor X | -4.41 | 0.70 | 3.00 | MSigDB Gene Set: V$MYCMAX_01 |
| E2F1 transcription factor | -7.33 | 1.01 | 3.94 | MSigDB Gene Set: V$E2F1_Q6_01 |
| E2F3 transcription factor | -5.73 | 0.69 | 3.84 | Ref. [1] |
| E2F1, TFDP1, RB1 transcription factors | -6.28 | 1.07 | 3.29 | MSigDB Gene Set: SGCGSSAAA_V$E2F1DP2_01 |
| E2F transcription factors | -4.18 | 1.52 | 2.69 | MSigDB Gene Set: V$E2F_01 |
| USF transcription factors | -3.92 | 0.55 | 2.99 | MSigDB Gene Set: V$USF_C |
| Genes with basally hypomethylated promoters upregulated by the combination of methylation and deacetylation inhibitors in ovarian carcinoma (CP70) cells | -3.09 | 1.38 | 2.58 | MSigDB Gene Set: TSADAC_HYPOMETH_OVCA_UP |
| c-fos serum response transcription factors | -3.35 | 0.98 | 3.22 | MSigDB Gene Set: CCAWWNAAGG_V$SRF_Q4 |
| ncRNA processing | -6.11 | 0.90 | 3.48 | GO: 0034470 |
| mRNA splicing | -3.24 | 0.39 | 3.03 | MSigDB Gene Set: MRNA_SPLICING |
| mRNA splicing complex | -2.55 | 0.34 | 2.66 | GO:0005681; MSigDB Gene Set: SPLICEOSOME |
| rRNA processing | -5.44 | 0.33 | 2.74 | GO: 0006364 |
| rRNA metabolic process | -5.40 | 0.32 | 2.77 | GO: 0016072 |
| Ribosome biogenesis | -6.46 | 0.67 | 3.80 | GO: 0042254 |
| Ribonucleoprotein complex | -4.20 | 0.59 | 3.10 | Ref: [2]; MSigDB Gene Set: RIBONUCLEOPROTEIN_COMPLEX |
| Nucleolus | -5.54 | 1.54 | 3.65 | GO: 0005730 |
| Nucleolus | -4.30 | 1.47 | 3.46 | GO: 0005730; MSigDB Gene Set: NUCLEOLUS |
| DNA recombination | -5.00 | 1.39 | 3.30 | GO: 0006310 |
| DNA recombination | -3.66 | 1.60 | 2.79 | Ref. [2]; MSigDB Gene Set: DNA_RECOMBINATION |
| DNA damage | -3.99 | 1.69 | 3.38 | MSigDB Gene Set: DNA_DAMAGE_SIGNALING |
| DNA repair | -2.98 | 1.01 | 2.76 | GO:0003684 |
| Pyrimidine metabolism | -4.56 | 1.56 | 3.12 | MSigDB Gene Set: PYRIMIDINE_METABOLISM |
| Pyrimidine metabolism | -4.27 | 0.77 | 2.60 | Ref. [3]; MSigDB Gene Set: HSA00240_PYRIMIDINE_METABOLISM |
| Nucleotidyl-transfer reaction | -3.63 | 1.39 | 2.93 | Ref. [2]; MSigDB Gene Set: NUCLEOTIDYLTRANSFERASE_ACTIVITY |
| Single-stranded DNA binding | -3.13 | 1.91 | 2.60 | GO:0003697 |
| G1/S cell cycle transition | -3.67 | 1.49 | 2.77 | MSigDB Gene Set: G1PATHWAY |
| Apoptosis | -3.16 | 1.69 | 2.43 | GO:0008632 |
| Genes that are upregulated in undifferentiated human embryonic stem cells | -3.01 | 1.13 | 2.55 | MSigDB Gene Set: BHATTACHARYA_ESC_UP |
| Rapamycin starvation-responsive genes | -5.01 | 1.06 | 4.27 | Ref. [4]; MSigDB Gene Set: PENG_RAPAMYCIN_DN |
| Genes that are down-regulated upon LPS stimulation in RAW 264.7 macrophages | -3.16 | 1.11 | 2.52 | MSigDB Gene Set: NEMETH_TNF_DN |
| Top genes down-regulated in MLL T-ALL | -4.95 | 1.49 | 3.43 | Ref. [5]; MSigDB Gene Set: FERRANDO_MLL_T_ALL_DN |
| Adhesion-related genes | -4.25 | 0.80 | 2.38 | Ref. [6]; MSigDB Gene Set: ASTIER_FN_DIFF |
| Cancer gene module 61 | -3.49 | 1.71 | 3.25 | Ref. [7]; Cancer gene module 61 |
| Cancer gene module 165 | -4.07 | 1.52 | 3.47 | Ref. [7]; Cancer gene module 165 |
| Cancer gene module 485 | -4.06 | 1.30 | 3.21 | Ref. [7]; Cancer gene module 485 |
| Genes that are up-regulated upon catechin treatment in MCF7 cells | -3.31 | 0.50 | 2.34 | Ref. [8,9] |
| Genes that are down-regulated upon sodium phenylbutyrate treatment in MCF7 cells | -3.29 | 1.18 | 2.37 | Ref. [8,9] |
| Genes that are up-regulated upon oxamic acid treatment in MCF7 cells | -2.87 | 1.08 | 2.97 | Ref. [8,9] |
| TNF-alpha-upregulated genes | -2.93 | 0.85 | 2.59 | MSigDB Gene Set: TNFALPHA_30MIN_UP |
| Neighborhood of protein tyrosine phosphatase, receptor type C (PTPRC) | 2.49 | -0.89 | -2.63 | MSigDB Gene Set: GNF2_PTPRC |
| Neighborhood of RAN, member RAS oncogene family | 2.60 | -0.88 | -3.12 | MSigDB Gene Set: GCM_RAN |
| Genes that are down-regulated by serum stimulation | 5.46 | -1.27 | -4.30 | Ref. [10]; MSigDB Gene Set: SERUM_FIBROBLAST_CORE_DN |
| Phosphoinositide binding | 2.40 | -0.38 | -2.77 | GO:0035091 |
| Homeobox A9 (HOXA9) and myeloid ecotropic viral integration site 1 (MEIS1) | 2.63 | -0.43 | -3.14 | MSigDB Gene Set: V$MEIS1AHOXA9_01 |
| Targets of microRNA miR-512-3P | 2.92 | -0.93 | -2.90 | MSigDB Gene Set: CAGCACT,MIR-512-3P |
| Genes that are up-regulated in the pubertal mouse mammary gland | 3.38 | 0.87 | -2.97 | MSigDB Gene Set: MAMMARY_DEV_UP |
| NRAS.vs.C3 down | 3.46 | -1.29 | -2.40 | Ref. [11] |
| TNF.2 down | 4.85 | -0.66 | -2.96 | Ref. [12] |

*^a^* t-statistics were obtained from three independent microarray experiments, and the scores in MEK1cr-expressing cells have *p* values greater than 0.005.

*^b^* MSigDB Gene Sets: <http://www.broadinstitute.org/gsea/msigdb/search.jsp>; GO (Gene Ontology): <http://www.geneontology.org/>; Cancer gene modules: <http://robotics.stanford.edu/~erans/cancer/browse_by_modules.html>
