# Supplementary material for: MEK2 Is Sufficient but Not Necessary for Proliferation and Anchorage-Independent Growth of SK-MEL-28 Melanoma Cells
Source: PLoS One. 2011 Feb 18;6(2):e17165. doi: 10.1371/journal.pone.0017165 (PMC3041822; doi:10.1371/journal.pone.0017165)
Supplement: Text S1 — Supplemental note (DOCX) [file pone.0017165.s006.docx]

**Supplemental Note**

The unexpected internal translation of non-tagged MEK when V5-MEK plasmids were transfected into mammalian cells was evidenced by the following observations. First, we constructed the EGFP sequence in the same expression vector and transfected cells with the V5-EGFP plasmid, and we also observed two immunoblotting signals (V5-EGFP and non-tagged EGFP) detected by EGFP antibodies. Second, after we removed the internal translation initiation codon (ATG) that was inserted with MEK sequences into the V5 expression vector during the construction process and transfected the modified plasmids into cells, we no longer observed the increase of non-tagged MEK expression.
