# Supplementary material for: MEK2 Is Sufficient but Not Necessary for Proliferation and Anchorage-Independent Growth of SK-MEL-28 Melanoma Cells
Source: PLoS One. 2011 Feb 18;6(2):e17165. doi: 10.1371/journal.pone.0017165 (PMC3041822; doi:10.1371/journal.pone.0017165)
Supplement: References S1 — Supplemental References (DOCX) [file pone.0017165.s007.docx]

**Supplemental References**

1. Bild AH, Yao G, Chang JT, Wang Q, Potti A, et al. (2006) Oncogenic pathway signatures in human cancers as a guide to targeted therapies. Nature 439: 353-357.

2. Ashburner M, Ball CA, Blake JA, Botstein D, Butler H, et al. (2000) Gene ontology: tool for the unification of biology. The Gene Ontology Consortium. Nat Genet 25: 25-29.

3. Kanehisa M, Araki M, Goto S, Hattori M, Hirakawa M, et al. (2008) KEGG for linking genomes to life and the environment. Nucleic Acids Res 36: D480-484.

4. Peng T, Golub TR, Sabatini DM (2002) The immunosuppressant rapamycin mimics a starvation-like signal distinct from amino acid and glucose deprivation. Mol Cell Biol 22: 5575-5584.

5. Ferrando AA, Armstrong SA, Neuberg DS, Sallan SE, Silverman LB, et al. (2003) Gene expression signatures in MLL-rearranged T-lineage and B-precursor acute leukemias: dominance of HOX dysregulation. Blood 102: 262-268.

6. Astier AL, Xu R, Svoboda M, Hinds E, Munoz O, et al. (2003) Temporal gene expression profile of human precursor B leukemia cells induced by adhesion receptor: identification of pathways regulating B-cell survival. Blood 101: 1118-1127.

7. Segal E, Friedman N, Koller D, Regev A (2004) A module map showing conditional activity of expression modules in cancer. Nat Genet 36: 1090-1098.

8. Lamb J, Crawford ED, Peck D, Modell JW, Blat IC, et al. (2006) The Connectivity Map: using gene-expression signatures to connect small molecules, genes, and disease. Science 313: 1929-1935.

9. Lamb J (2007) The Connectivity Map: a new tool for biomedical research. Nat Rev Cancer 7: 54-60.

10. Chang HY, Sneddon JB, Alizadeh AA, Sood R, West RB, et al. (2004) Gene expression signature of fibroblast serum response predicts human cancer progression: similarities between tumors and wounds. PLoS Biol 2: E7.

11. Whitwam T, Vanbrocklin MW, Russo ME, Haak PT, Bilgili D, et al. (2007) Differential oncogenic potential of activated RAS isoforms in melanocytes. Oncogene 26: 4563-4570.

12. Tian B, Nowak DE, Jamaluddin M, Wang S, Brasier AR (2005) Identification of direct genomic targets downstream of the nuclear factor-kappaB transcription factor mediating tumor necrosis factor signaling. J Biol Chem 280: 17435-17448.
